# Dissecting the Genetic Structure of Maize Leaf Sheaths at Seedling Stage by Image-Based High-Throughput Phenotypic Acquisition and Characterization

**DOI:** 10.3389/fpls.2022.826875

**Published:** 2022-06-28

**Authors:** Jinglu Wang, Chuanyu Wang, Xianju Lu, Ying Zhang, Yanxin Zhao, Weiliang Wen, Wei Song, Xinyu Guo

**Affiliations:** ^1^Beijing Key Lab of Digital Plant, Information Technology Research Center, Beijing Academy of Agriculture and Forestry Sciences, Beijing, China; ^2^National Engineering Research Center for Information Technology in Agriculture, Beijing Academy of Agriculture and Forestry Sciences, Beijing, China; ^3^Beijing Key Laboratory of Maize DNA Fingerprinting and Molecular Breeding, Maize Research Center, Beijing Academy of Agriculture and Forestry Sciences, Beijing, China; ^4^Key Laboratory of Crop Genetics and Breeding of Hebei Province, Institute of Cereal and Oil Crops, Hebei Academy of Agriculture and Forestry Sciences, Shijiazhuang, China

**Keywords:** maize, leaf sheath, image-based traits, GWAS, pathways

## Abstract

The rapid development of high-throughput phenotypic detection techniques makes it possible to obtain a large number of crop phenotypic information quickly, efficiently, and accurately. Among them, image-based phenotypic acquisition method has been widely used in crop phenotypic identification and characteristic research due to its characteristics of automation, non-invasive, non-destructive and high throughput. In this study, we proposed a method to define and analyze the traits related to leaf sheaths including morphology-related, color-related and biomass-related traits at V6 stage. Next, we analyzed the phenotypic variation of leaf sheaths of 418 maize inbred lines based on 87 leaf sheath-related phenotypic traits. In order to further analyze the mechanism of leaf sheath phenotype formation, 25 key traits (2 biomass-related, 19 morphology-related and 4 color-related traits) with heritability greater than 0.3 were analyzed by genome-wide association studies (GWAS). And 1816 candidate genes of 17 whole plant leaf sheath traits and 1,297 candidate genes of 8 sixth leaf sheath traits were obtained, respectively. Among them, 46 genes with clear functional descriptions were annotated by single nucleotide polymorphism (SNPs) that both Top1 and multi-method validated. Functional enrichment analysis results showed that candidate genes of leaf sheath traits were enriched into multiple pathways related to cellular component assembly and organization, cell proliferation and epidermal cell differentiation, and response to hunger, nutrition and extracellular stimulation. The results presented here are helpful to further understand phenotypic traits of maize leaf sheath and provide a reference for revealing the genetic mechanism of maize leaf sheath phenotype formation.

## Introduction

Maize leaf sheath is located at the base of leaf and wraps around the stem node. It plays a role of protecting and supporting the leaf. At the same time, it can protect the young and tender intermediate meristems and young buds on the stem, and enhance the mechanical support of the stem (Dong et al., 2019). In the sink-source relationship, the leaf sheath can be used as a nutrient storage organ in the early stage, namely, “sink.” And it can be also used as an organ for the production or export of assimilates in the later stage of growth, that is, “source.” It is well known that leaf sheaths usually have elongation zones. As a result of intercellular growth, the cells elongate in two separate directions, above and below, and differentiate into longitudinally parallel vascular bundles (Russell and Evert, 1985). Hence, maize leaf sheaths can also be used as part of the “flow.” In summary, the role of maize leaf sheaths in the plant is very important and deserves more attention and in-depth study. In addition, maize purple plant pigments are anthocyanin pigments. A large number of domestic and foreign studies have shown that purple-red anthocyanin pigments have anti-oxidation, anti-aging, immune enhancement and tumor prevention functions (Zhang et al., 2014; Li et al., 2020; Peniche-Pavia and Tiessen, 2020; Chatham and Juvik, 2021). Therefore, it is of great theoretical and practical importance to study the phenotypic characteristics of maize leaf sheaths and to analyze their genetic structure.

Maize has a rich diversity due to its long planting history and wide geographical span. Among them, the leaf sheath phenotype of maize also varies between populations, particularly in color. As a consequence, traditional studies of maize leaf sheaths are usually based on a qualitative description or classification of leaf sheath color. The leaf sheaths of maize commonly come in purple and green. It has been pointed out that these color changes are usually related to pigments (Fan et al., 2008). Li et al. (2018) conducted genetic analysis and gene localization for the purple leaf sheath trait using a recombinant inbred line population of maize and found that the gene *GRMZM5G822829* was highly significantly differentially expressed between the purple and green leaf sheath parents. Yang S. et al. (2014) used the maize white sheath inbred line K10 as the research material, and conducted preliminary genetic mechanism and gene mapping of the white sheath traits. The results showed that the white leaf sheath trait has nothing to do with cytoplasmic inheritance, but was controlled by recessive nuclear genes and was under polygenic control.

Nowadays, with the rapid development of high-throughput phenotyping technology, it has become possible to obtain massive crop phenotypic information quickly, efficiently and accurately (Zhao et al., 2019). Among them, image-based phenotype acquisition methods have been widely used in crop phenotype identification and characterization due to their automatic, non-invasive, non-destructive, and high-throughput characteristics (Green et al., 2012; Tanabata et al., 2012; Bucksch et al., 2014; Gage et al., 2017; Chopin et al., 2018; Zhang et al., 2020; Zhou et al., 2021). Based on image data, a variety of phenotypes can be analyzed, which can break through the limitations of subjective cognition and carry out deeper research (Mir et al., 2019). For example, the web-based tool PhenoPhyte is a flexible affordable method to quantify 2D phenotypes from imagery. And it can distinguish different experimental Settings through experimental database management and calculate the phenotypic parameters related to leaf area in phenotypic images (Green et al., 2012). SmartGrain, as a high-throughput phenotyping software for measuring seed shape through image analysis, using a new image analysis method to reduce the time taken in the preparation of seeds and in image capture (Tanabata et al., 2012). TIPS is a system for automated image-based phenotyping of maize tassels, and it allows morphological features of maize tassels to be quantified automatically, with minimal disturbance, at a scale that supports population-level studies. And it is expected to accelerate the discovery of associations between genetic loci and tassel morphology characteristics (Gage et al., 2017). Another maize image analysis software is Maize-IAS, which is an integrated application supporting one-click analysis of maize phenotype, embedding multiple functions, with a high efficiency and potential capability to image-based plant research (Zhou et al., 2021). Thus, image technology has become a high-throughput means to obtain and analyze the phenotypic information of large populations of crops. The phenotypic information can be used for quantitative trait loci mapping and genome-wide association studies. It is helpful to break the gap between crop traits and genetic markers and promote the study of crop phenotypic-genotype association (Zhao et al., 2019; Yang et al., 2020; Song et al., 2021).

Genome-wide association study (GWAS) as an analytical method for identifying the relationship between a target trait and a genetic marker or candidate gene within a group of individuals, provides a powerful tool for researchers concerned with and exploring the genetic mechanisms of phenotype formation across multiple individuals (Xiao et al., 2016; Liu and Yan, 2019). In particular, the mixed linear model (MLM) methods have proven useful in controlling for population structure and relatedness within GWAS. In the MLM-based methods, population structure is fitted as a fixed effect, while kinship among individuals is incorporated into the variance-covariance structure of individual random effects (Zhang et al., 2010). Since the publication of maize B73 reference genome (Schnable et al., 2009), GWAS has been widely used in maize genetics research, and has played a great role in the analysis of genetic mechanisms such as traditional maize agronomic traits (Wallace et al., 2016; Zhou et al., 2016; Li et al., 2017; Dai et al., 2018; Du et al., 2018; Zhou et al., 2018; Owens et al., 2019), key phenotypes (Cui et al., 2016; Li et al., 2016; Liu et al., 2016; Zhang et al., 2016a; Sanchez et al., 2018; Guo et al., 2019; Mazaheri et al., 2019) and stress resistance (Zhang et al., 2016b; Shi et al., 2018; Cooper et al., 2019; Wang et al., 2019; Xie et al., 2019). However, there are few genetic studies on the phenotype of maize leaf sheath.

**FIGURE 1:**
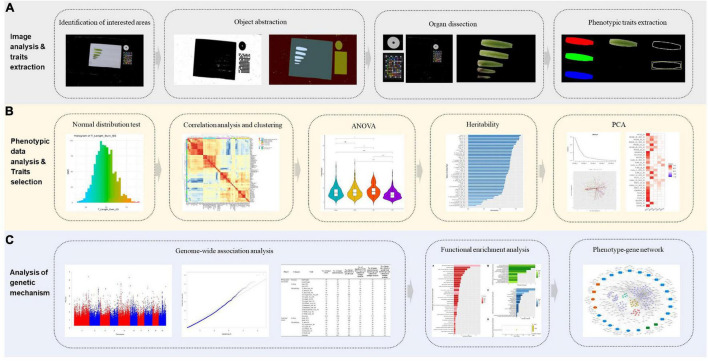
The flowchart of image-based high-throughput phenotypic analysis and genetic mechanism analysis of maize leaf sheaths at seedling stage. **(A)** Image analysis and phenotypic traits extraction. **(B)** Phenotypic data analysis and key traits selection for genetic analysis. **(C)** Genetic mechanism analysis of key traits.

Through image acquisition, image segmentation, feature extraction and manual measurement of leaf sheaths of 418 maize inbred lines at V6 stage, this study proposed a method to define and analyze the shape, size, color and other phenotypes related to leaf sheaths, and developed a pipeline for image-based traits with phenotypic data analysis and genetic mechanism analysis (Figure 1). In addition, 87 leaf sheath-related phenotypic traits including morphology, color and biomass were obtained. Based on these phenotypic traits, leaf sheath characteristics of maize association analysis population were analyzed. In order to further analyze the mechanism of leaf sheath phenotype formation, 25 key traits of maize were analyzed by GWAS, and 1,816 candidate genes of 17 whole plant leaf sheath traits and 1,297 candidate genes of 8 sixth leaf sheath traits were obtained, respectively. This study has achieved high-throughput acquisition of the phenotype from maize leaf sheath. And it also can provide a reference for revealing the genetic mechanism of maize leaf sheath phenotype formation.

## Materials and Methods

### Plant Materials, Growth Conditions, and Sample Collection

418 inbred lines used in this study were from the maize association mapping panel published by Yang et al. (2011); Supplementary Table 1, which were classified into four subpopulations: Non-stiff stalk (NSS) with 124 lines, Stiff stalk (SS) with 31 lines, Tropical-subtropical (TST) with 164 lines, and 99 mixed lines (Mixed). The plants were grown in the Beijing Academy of Agriculture and Foresting Science in Beijing, China. Maize seeds were planted manually at a depth of 5 cm on 17 May 2019. Each inbred line was planted in 4 rows with 7 plants per row. Planting density and water and fertilizer management were based on local field production (Lu et al., 2020).

### Image Acquisition, Analysis, and Feature Extraction

Maize plants were grown to the V6 stage, and three plants were sampled from each inbred line population. The leaf sheaths were spread out on a white soft background plate and fixed with pins. Blade images were captured by an image acquisition device (Canon EOS 5D Mark III) with a resolution of 5,760 × 3,840 pixels. The image processing program (Figure 1A) is developed by Visual Studio Express 2015, using the open-source image processing library OpenCV 2.3.

The image processing and feature extraction methods were summarized as follows: (**a) Identification of interested areas.** The original color image was converted into a grayscale image, an adaptive thresholding algorithm was used to segment foreground and background. (**b) Object abstraction.** The foreground contained a circular marker, a color checker board and leaf sheaths, these components were separated according to shape, inner composition pattern and chromatic property. (**c) Organ dissection.** The largest contour was considered as the sixth leaf sheath, the rest contours were labeled as the fifth leaf sheath candidate regions. First, found out bounding box of these candidate regions, and calculated length/width ratio, if the ratio value was more than 3.0, then we argued that belonged to leaf sheath candidate. Chosen the largest leaf sheath candidate regions to compute centroid coordinate which denote by *C*_*cdit*_, and centroid of sixth leaf sheath denoted by *C*_*six*_, the Euclidean distance of above two point is *D*_*cent*_, if the *D*_*cent*_ was less than 1/2 length of sixth leaf sheath bounding box, the candidate regions was labeled as the fifth leaf sheath, repeated the procedure for the remaining candidates, until the last one was tested. (**d) Phenotypic traits calculation.** Phenotypic measurement and image-based feature extraction were performed on the whole plant leaf sheath and the sixth leaf sheath of maize at the V6 stage, respectively. The specific calculation and definitions for each trait are detailed in Supplementary Table 2 and Supplementary Note 1.

Together with the two biomass traits, dry weight (DryWeight) and fresh weight (FreshWeight) of the whole plant, measured manually by electronic balances, totally 87 leaf sheaths related traits that covering three types (morphology, color and biomass) and two objects (the whole plant leaf sheath and the sixth leaf sheath) were obtained in this study (Supplementary Table 2).

### Statistical Analysis of Phenotypic Data

The “lm” function in R (Version 3.6.3) software^1^ was used to carry out linear regression analysis on the leaf sheath area extracted by the image-based method and the dry/fresh weight measured by manual. The *R*^2^ obtained from the model represent the accuracy of the software algorithm.

Analysis of variance (ANOVA) and descriptive statistical analysis were conducted via R (Version 3.6.3) software to determine whether each phenotype is different between different subpopulations. Pearson correlation analysis was used to calculate the correlation coefficients among phenotypic traits. And *pamk*, a function of R package “FPC,” was used to perform unsupervised hierarchical cluster analysis (HCA) using Pearson correlation coefficient as distance measure, and then 87 traits were grouped based on clustering.

Broad sense heritability (H^2^) usually means the percentage of genetic variation (*V*_*A*_) to the total variation of a phenotype. It can be used to compare the relationship between genetic ($\sigma_{A}^{2})$ and environmental ($\sigma_{e}^{2})$ factors for a specific phenotypic variation (*V*_*P*_). Heritability (H^2^) was calculated for each trait as follows:


$$H^{2}=\frac{V_{A}}{V_{P}}=\frac{\sigma_{A}^{2}}{\sigma_{A}^{2}+\sigma_{e}^{2}}$$

where $\sigma_{A}^{2}$ is the genetic variance, $\sigma_{e}^{2}$ is the environmental variance. The analysis was performed in ASReml-R v.4.0 by using the “asreml” function of R package asreml (Butler, 2009).

### Genome-Wide Association Study

Genotypic data of maize association mapping panel were obtained from Maizego.^2^ Firstly, the genotypic data of 418 inbred lines needed in this study were extracted, and 794,722 SNPs with minimum allele frequency (MAF) greater than 0.05 and call rate greater than 0.9 filtered by PLINK 1.09 software were used in GWAS. For GWAS, a multi-locus random-SNP-effect mixed linear model tool (R package “mrMLM” version 4.0) (Zhang et al., 2019) including six multi-locus GWAS methods (mrMLM, FASTmrMLM, FASTmrEMMA, ISIS EM-BLASSO, pLARmEB, and pKWmEB) was used on each leaf sheath related phenotypic traits separately to test the statistical association between phenotypes and genotypes. In addition, population structure estimated by STRUCTURE program version 2.3.4 (Hubisz et al., 2009) and relative kinship calculated by TASSEL 5 (Bradbury et al., 2007) with 794,722 SNPs were brought into the model. These six Multi-locus GWAS methods were processed in two steps. First, each SNP on the genome was filtered with a *P*-value ≤ 0.5/N, N is the total number of genome-wide SNPs. Then, all the SNPs that are potentially associated with the trait were included in a multi-locus genetic model further screened with a defeat *P*-value = 0.0002 to declare a significance of SNPs that associated with a given trait. The results obtained by the six multi-locus GWAS methods were regarded as significant SNPs associated with phenotypic traits. Furthermore, SNPs with the highest significance obtained by each method were regarded as Top 1, and SNPs identified by multiple methods were considered to be more reliable results. All candidate genes were annotated by ANNOVAR software according to the latest maize B73 reference genome (B73 RefGen_v4) available in EnsemblPlants^3^ and NCBI Gene database.^4^

### Functional and Network Analysis

The biological functions of candidate genes with high confidence for each phenotypic trait (Top1 SNP annotation or multiple GWAS validation) were explored by pathway enrichment analysis. Enrichment analysis of Gene Ontology (GO) (Ashburner et al., 2000) was conducted using PlantRegMap (Jin et al., 2015). And KOBAS V3.0 (Bu et al., 2021) was used to enrich Kyoto Encyclopedia of Genes and Genomes (KEGG) (Kanehisa, 2002) pathway. Among them, GO terms and KEGG pathways with the *P*-value less than 0.05 were considered to be significantly enriched results.

In order to have a better view of the relationship between each trait and its candidate genes, an open-source software platform (Cytoscape v3.7.2) (Shannon et al., 2003) was used to visualize the complex trait-candidate gene-pathway network and integrate the input data by their attribute information.

## Results

### Phenotypic Extraction of Leaf Sheath

In this study, image analysis was used to replace the traditional leaf sheath phenotype acquisition methods. In addition to conventional traits such as length, width, and surface area of leaf sheaths, many traits such as leaf sheaths morphology and color were also extracted based on image, realizing high-throughput acquisition of phenotypic of maize leaf sheaths. After processing the original image, a total of 1,116 valid image samples were obtained, covering 418 inbred lines. According to these leaf sheath images, the characteristics of the whole plant leaf sheath and the sixth leaf sheath of maize at V6 stage were extracted, and a total of 85 2D leaf sheath-related traits were obtained. Together with two biomass traits obtained by measuring the dry and fresh weight of the whole plant leaf sheath, totally 87 traits covering morphology, color and biomass these three types (Supplementary Table 2 and Supplementary Note 1) were analyzed in this study. Of these, there were 50 whole plant leaf sheath traits, including two biomass traits, 18 morphological traits and 30 color traits. And 37 traits of the sixth leaf sheath, including 7 morphological traits and 30 color traits.

**FIGURE 2:**
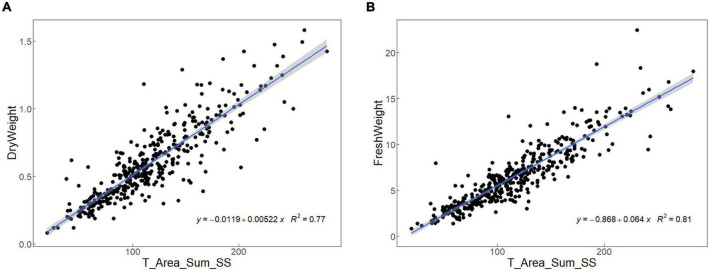
The R^2^ obtained from the linear regression analysis on the leaf sheath area extracted by the image-based method and the dry **(A)**/fresh **(B)** weight measured by manual.

The measurement accuracy of the image-based phenotypic acquisition method was valued by the linear regression analysis on the leaf sheath area extracted by the image-based method and the dry/fresh weight measured by manual, and the *R*^2^ obtained from the model represent the accuracy of the software algorithm. As shown in Figure 2, the *R*^2^ of two models were 0.77 and 0.81, respectively. The *R*^2^ of both models were close to 1, indicating that the measurement accuracy of the image-based phenotypic acquisition method is high, and the traits could be used for subsequent analysis.

### Phenotypic Characteristics of Leaf Sheath

The basic statistical analysis results (Supplementary Table 3) of 87 leaf sheath traits showed that the phenotypic traits of inbred lines in maize association analysis population had extensive continuous variation, with the variation coefficient ranging from –0.67 to 21.49. Furthermore, it can be seen from the data histogram that the phenotypic traits data were normally distributed, indicating that all traits were quantitative traits.

**FIGURE 3:**
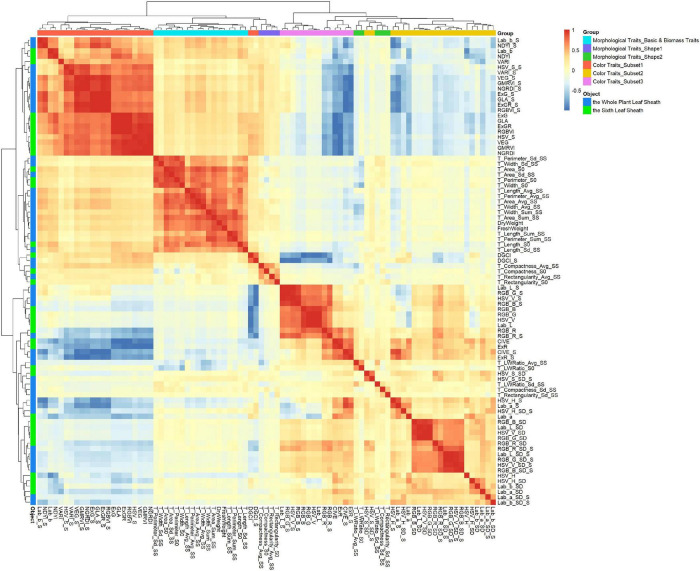
Correlation analysis and clustering of 87 leaf sheath traits. 87 traits were obtained from two objects: the whole plant leaf sheath and the sixth leaf sheath. After clustering, 87 traits were divided into six groups and marked with different colors: Morphological Traits_Basic and Biomass Traits, Morphological Traits_Shape1, Morphological Traits_Shape2, Color Traits_Subset1, Color Traits_Subset2 and Color Traits_Subset3.

Pearson correlation analysis was performed on 87 leaf sheath phenotypes, and clustering was performed based on Pearson correlation coefficient, as shown in Figure 3. Cluster analysis results showed that 87 phenotypes of the three types could be divided into 6 groups, and each group had clear characteristics (marked with different colors in Figure 3). The Morphological characteristics of leaf sheath can be divided into three groups. Group I (Morphological Traits_Basic): 16 basic morphological traits describing leaf sheath length, width and area, etc. Group II (Morphological Traits_Shape1): 4 traits were used to describe the morphological shape of leaf sheath. And Group III (Morphological Traits_Shape2): 5 traits to characterize the variation of morphological type of leaf sheath. There was no significant correlation between the 9 traits describing leaf sheath shape in the two groups and other traits, indicating that leaf sheath shape was basically unrelated to leaf sheath size, area and color. The 16 basic morphological traits of leaf sheath had a significant positive correlation with DryWeight and FreshWeight (*P*-value < 0.05), and clustered into the same group (Morphological Traits_Basic and Biomass Traits). This result is consistent with prior knowledge, which indicates the reliability of data and the significance of obtaining various traits from images. The leaf sheath Color Traits were also divided into three groups. The first group (Color Traits_Subset1) consisted mainly of comprehensive color traits, the second group (Color Traits_Subset2) of traits were mostly the variation degree of the single-channel color values, and the third group (Color Traits_Subset3) was composed of single-channel color traits and four comprehensive color traits. The 24 comprehensive color traits were separated into two groups, because CIVE, CIVE_S, ExR, and ExR_S mainly represent red, while the other comprehensive traits mainly represent blue and green, indicating the accuracy of data extraction.

**FIGURE 4:**
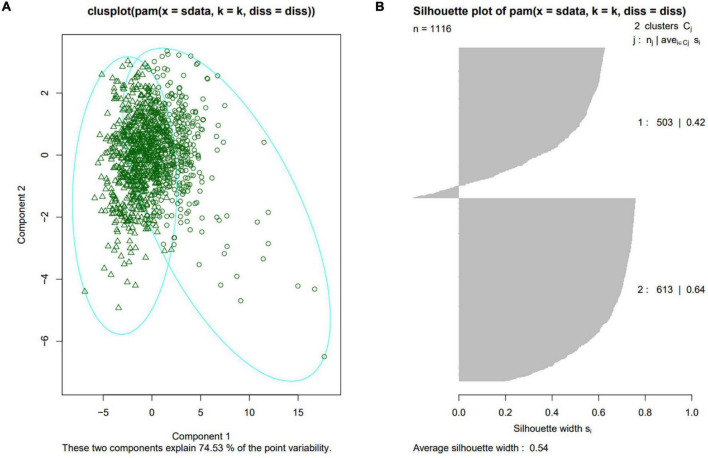
Sample grouping results based on 12 morphological traits of the whole plant leaf sheath. **(A)** Clusplot of the first two principal components of 12 morphological traits of the whole plant leaf sheath. **(B)** Silhouette plot of samples characterized by 12 morphological traits of the whole plant leaf sheath.

87 phenotypic traits were analyzed among different subpopulations in turn. The results showed that the inbred lines of TST subpopulation had distinct characteristics and were significantly different from at least one subpopulation in 84 traits (96.55%) (*P*-value < 0.05). Among them, 64 traits (73.56%) showed significant differences between TST and all other three subpopulations (*P*-value < 0.05) (Supplementary Figure 1). The traits with significant differences between TST and other subpopulations covered all three types of traits, indicating that the leaf sheaths of tropical and subtropical maize inbred lines (TST) were different from those of other climate zone maize inbred lines in terms of morphology, color and biomass. In order to further explore which traits had the greatest difference between TST and other subpopulations, excluding the two biomass traits, the other 62 phenotypic traits were divided into four groups according to trait types and research objects. Consequently, the four groups were 12 leaf sheath morphological traits of whole plant, 21 leaf sheath color traits of whole plant, 4 leaf sheath morphological traits and 25 leaf sheath color traits of the sixth leaf, respectively. Then principal component analysis (PCA) was carried out for each group of traits, and the results showed that the samples analyzed in each group were divided into two categories (Figure 4A and Supplementary Figure 2). However, the Average Silhouette Width is the highest after clustering according to 12 morphological traits of the whole plant leaf sheath, which is 0.54 (Figure 4B). In addition, 10 of the 12 traits (T_Area_Avg_SS, T_Area_Sd_SS, T_Area_Sum_SS, T_Compactness_Avg_SS, T_Length_Avg_SS, T_Width_Avg_SS, T_Length_Sd_SS, T_Width_Sd_SS, T_LWRatio_Avg_SS, T_Width_Sum_SS, T_Perimeter_Avg_SS, T_Perimeter_Sd_SS) were basic morphological traits of leaf sheath morphology, suggesting that the main differences between TST and other subpopulations were manifested in the conventional phenotypic traits such as leaf sheath length, width and area.

### Heritability Analysis

**FIGURE 5:**
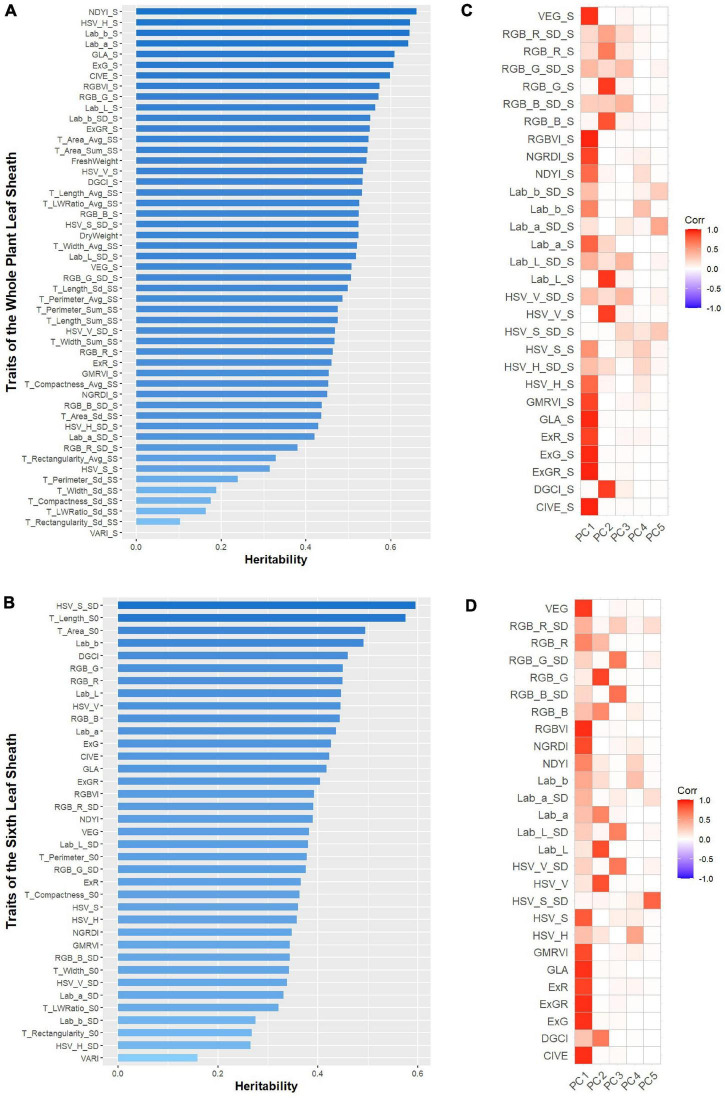
The broad-sense heritability (H^2^) of the investigated 87 phenotypic traits and principal component analysis (PCA) of color traits with heritability greater than 0.3. **(A)** The broad-sense heritability (H^2^) of the 50 phenotypic traits of the whole plant leaf sheath. **(B)** The broad-sense heritability (H^2^) of the 37 phenotypic traits of the sixth leaf sheath. **(C)** The first five principal components for color traits of the whole plant leaf sheath. **(D)** The first five principal components for color traits of the sixth leaf sheath.

Heritability analysis was performed on 87 leaf sheath phenotypic traits extracted from 2D images, and the results are shown in Figures 5A,B. For the whole plant leaf sheath traits, the heritability of these 50 traits ranged from 1.01E-07 to 0.6601. Among them, the heritability of DryWeight and FreshWeight was 0.5234 and 0.5429, respectively. And the heritability of 18 morphological traits ranged from 0.1029 to 0.5470, and 13 (72.22%) of these traits had a heritability greater than 0.3. Except VARI-S, the heritability of the other 29 color traits ranged from 0.3142 to 0.6601, and 29 (96.67%) of these color traits had a heritability greater than 0.3. For the sixth leaf sheath traits, the heritability of these 37 traits ranged from 0.1594 to 0.5754. And the heritability of 7 morphological traits ranged from 0.2683 to 0.5754, of which 6 (85.71%) had heritability greater than 0.3. The heritability of 30 color traits ranged from 0.1594 to 0.5955, and 27 (90.00%) of them had heritability greater than 0.3. To further investigate the genetic mechanism of phenotypic traits related to maize leaf sheaths, traits with heritability greater than 0.3 were screened for further genetic analysis in this study.

However, due to the large number of color traits with heritability greater than 0.3, PCA was applied to the color traits of the whole plant and the sixth leaf sheath separately to accomplish the dimensionality reduction and key feature extraction. The results showed that for the color traits of these two objects, the first and second principal components (PCs) were strongly correlated with most color variables, and the cumulative contribution value of the first two principal components was 0.72 and 0.66, respectively (Figures 5C,D). Therefore, 4 traits that consisted of the first two PCs of the two objects color traits were selected for subsequent GWAS. Adding to the 21 non-color traits with heritability greater than 0.3, totally 25 key traits (2 biomass-related, 19 morphology-related and 4 color-related traits) with high heritability was used to explore the genetic mechanisms by GWAS.

### Significant Single Nucleotide Polymorphism Obtained by Genome-Wide Association Study

In conclusion, the multi-locus random-SNP effect mixed linear model in R software package “mrMLM” (version 4.0) (Zhang et al., 2019) was used for GWAS analysis of biomass traits, morphological traits and color principal components related to 2D leaf sheaths, including 17 whole plant leaf sheath traits and 8 sixth leaf sheath traits. Finally, 1142 SNPs significantly related to 17 whole plant leaf sheath traits and 755 SNPs significantly related to 8 sixth leaf sheath traits were identified (*P*-value < 6.4e-07) (Table 1). Additionally, among the results of the 6 GWAS methods, the most significant (Top1) SNP obtained by each method and the SNPs verified by two or more methods were considered to be highly reliable results. As a consequence, 152 SNPs significantly associated with 17 whole plant leaf sheath traits and 85 SNPs significantly associated with 8 sixth leaf sheath traits were obtained. These highly significant or multi-method verification results will be reported as the key findings of this study.

**TABLE 1:** Summary of significant loci from genome-wide association study.

Object	Category	Trait	No. of unique SNPs	No. of unique annotated genes	No. of genes only related to specific trait	No. of significant SNPs listed Top 1* and validated by multiple methods	No. of unique annotated genes listed Top1 and validated by multiple methods	No. of genes only related to specific trait listed Top 1 and validated by multiple methods
Whole plant leaf sheath	Biomass	DryWeight	35	62	26	6	12	12
		Freshweight	47	86	49	10	19	17
	Color	Sum_PC1	58	105	79	11	22	16
		Sum_PC2	75	132	101	9	18	18
	Morphology	T_Area_avg_SS	43	72	26	10	19	14
		T_Area_Sd_SS	46	77	25	10	16	8
		T_Area_sum_SS	41	71	22	12	24	14
		T_Compactness_Avg_SS	213	348	242	11	19	17
		T_Length_Avg_SS	53	94	50	9	18	14
		T_Length_Sd_SS	34	59	36	9	15	10
		T_Length_Sum_SS	50	87	50	12	21	17
		T_LWRatio_Avg_SS	38	74	54	9	18	18
		T_Perimeter_Avg_SS	35	65	30	8	14	7
		T_Perimeter_Sum_SS	48	91	46	13	26	16
		T_Rectangularity_Avg_SS	347	591	456	12	21	17
		T_Width_Avg_SS	31	62	27	5	10	6
		T_Width_Sum_SS	45	84	25	7	14	8
		**Summary**	**1,142**	**1,816**	**1,344**	**152**	**275**	**229**
Sixth leaf sheath	Color	Sixth_PC1	75	134	102	12	22	18
		Sixth_PC2	269	478	400	11	21	21
	Morphology	T_Area_S0	45	77	25	10	18	6
		T_Compactness_S0	72	122	85	13	21	19
		T_Length_S0	53	95	42	14	23	19
		T_LWRatio_S0	136	245	192	11	18	16
		T_Perimeter_S0	48	83	26	11	18	12
		T_Width_S0	84	154	97	9	16	12
		**Summary**	**755**	**1,297**	**969**	**85**	**146**	**123**

*Top1: the most significant SNP obtained by each GWAS method.

### Identification and Annotation of Candidate Genes

Gene annotation was performed on 1142 SNPs significantly related to 17 whole plant leaf sheath traits and 755 SNPs significantly related to 8 sixth leaf sheath traits by using the latest maize B73 reference genome (B73 RefGen_v4) available in EnsemblPlants and NCBI Gene databases. Finally, 1,816 candidate genes of 17 whole plant leaf sheath traits and 1,297 candidate genes of 8 sixth leaf sheath traits were obtained, respectively. Among them, 275 genes of 17 whole plant leaf sheath traits and 146 genes of 8 sixth leaf sheath traits were derived from the most significant SNP (Top1) obtained by each method and SNPs annotations verified by multiple methods (Table 1). Genes annotated by SNPs with the highest significance or multi-method validation were further retrieved in NCBI Gene database, and 270 candidate genes of 25 key traits for leaf sheath phenotype had detailed functional descriptions (Supplementary Table 4). Among them, a total of 46 genes with clear functional descriptions were annotated by SNPs that both Top1 and multi-method validated (Table 2).

**TABLE 2:** Detailed functional descriptions of 46 genes annotated by both Top1 and multi-method validated SNPs.

Gene	Description	Chromosome	Genomic_ nucleotide_ accession.version	Start_position_ on_the_genomic_ accession	End_position_on _the_genomic_ accession	Trait	Object
GRMZM2G073826	Transcription factor MYB3R-5	5	NC_050100.1	137,986,909	138,015,364	FreshWeight	Whole plant leaf sheath
GRMZM2G418206	Proteinaceous RNase P 1, chloroplastic/mitochondrial	5	NC_050100.1	137,893,158	137,908,282	FreshWeight	Whole plant leaf sheath
GRMZM2G040452	Catalytic/protein phosphatase type 2C	4	NC_050099.1	237,957,682	237,960,493	Sum_PC1	Whole plant leaf sheath
GRMZM2G085945	Zinc finger protein	5	NC_050100.1	219,927,636	219,928,886	Sum_PC2	Whole plant leaf sheath
Zm00001d009690	RNA cytidine acetyltransferase 1	8	NC_050103.1	76,264,531	76,273,801	Sum_PC2	Whole plant leaf sheath
GRMZM2G103721	Phosphatidylinositol 3-kinase, root isoform	4	NC_050099.1	74,538,889	74,548,829	T_Area_Avg_SS	Whole plant leaf sheath
GRMZM2G134248	Long chain base biosynthesis protein 1a	4	NC_050099.1	74,702,902	74,704,363	T_Area_Avg_SS	Whole plant leaf sheath
GRMZM2G156238	C2 Domain-containing protein At1g53590	4	NC_050099.1	226,930,981	226,946,730	T_Area_Avg_SS	Whole plant leaf sheath
GRMZM2G126860	Protein SUPPRESSOR OF K(+) TRANSPORT GROWTH DEFECT 1	8	NC_050103.1	14,003,169	14,008,552	T_Area_Avg_SS	Whole plant leaf sheath
GRMZM2G126956	DNA damage-binding protein 2	8	NC_050103.1	14,023,075	14,027,906	T_Area_Avg_SS	Whole plant leaf sheath
GRMZM2G161169	Taxane 10-beta-hydroxylase	4	NC_050099.1	6,214,120	6,216,528	T_Area_Sum_SS	Whole plant leaf sheath
GRMZM2G065496	B3 Domain-containing protein	1	NC_050096.1	168,954,911	168,957,922	T_Compactness_ Avg_SS	Whole plant leaf sheath
zma-MIR169i	MicroRNA MIR169i	4	NC_050099.1	49,606,834	49,607,024	T_Compactness_ Avg_SS	Whole plant leaf sheath
FHA9	Myosin-9	1	NC_050096.1	5,773,058	5,778,341	T_Length_Avg_SS	Whole plant leaf sheath
GRMZM2G371137	Probable LRR receptor-like serine/threonine-protein kinase At1g12460	1	NC_050096.1	5,836,567	5,841,667	T_Length_Avg_SS	Whole plant leaf sheath
GRMZM2G047715	Homeobox-leucine zipper protein HOX7	4	NC_050099.1	126,441,242	126,446,006	T_Length_Avg_SS	Whole plant leaf sheath
GRMZM2G105933	Putative protein kinase superfamily protein	4	NC_050099.1	126,356,761	126,359,205	T_Length_Avg_SS	Whole plant leaf sheath
GRMZM2G097605	DNA repair helicase UVH6	10	NC_050105.1	91,197,531	91,202,542	T_Length_Sd_SS	Whole plant leaf sheath
GRMZM2G135770	Putative regulator of chromosome condensation (RCC1) family protein	4	NC_050099.1	84,989,713	84,995,057	T_Length_Sum_SS	Whole plant leaf sheath
GRMZM2G419305	Agenet domain-containing protein/bromo-adjacent homology (BAH) domain-containing protein	4	NC_050099.1	85,143,298	85,148,546	T_Length_Sum_SS	Whole plant leaf sheath
GRMZM2G030839	Phosphomevalonate kinase	9	NC_050104.1	148,330,626	148,338,890	T_LWRatio_ Avg_SS	Whole plant leaf sheath
GRMZM2G094592	IRK-interacting protein	7	NC_050102.1	138,421,602	138,423,876	T_Perimeter_ Avg_SS	Whole plant leaf sheath
GRMZM2G143160	Serine/threonine-protein kinase MPS1	1	NC_050096.1	271,894,088	271,899,733	T_Perimeter_ Sum_SS	Whole plant leaf sheath
GRMZM2G147332	Oxysterol-binding protein-related protein 1C	1	NC_050096.1	271,997,558	272,015,271	T_Perimeter_ Sum_SS	Whole plant leaf sheath
GRMZM2G319357	Low molecular weight protein-tyrosine-phosphatase slr0328	1	NC_050096.1	209,876,521	209,881,331	T_Perimeter_ Sum_SS	Whole plant leaf sheath
GRMZM2G022926	OSJNBa0070C17.17-like protein	10	NC_050105.1	144,286,874	144,289,145	T_Perimeter_ Sum_SS	Whole plant leaf sheath
GRMZM2G028676	Vacuolar ATPase assembly integral membrane protein VMA21-like domain	10	NC_050105.1	144,223,404	144,224,474	T_Perimeter_ Sum_SS	Whole plant leaf sheath
GRMZM2G128248	dnaJ protein	8	NC_050103.1	172,072,905	172,075,250	T_Rectangularity_ Avg_SS	Whole plant leaf sheath
GRMZM2G123537	Pumilio homolog 3	4	NC_050099.1	176,122,607	176,128,541	T_Width_Sum_SS	Whole plant leaf sheath
zma-MIR172c	MicroRNA MIR172c	4	NC_050099.1	176,265,726	176,265,848	T_Width_Sum_SS	Whole plant leaf sheath
GRMZM2G309025	S-domain class receptor-like kinase 3	7	NC_050102.1	165,446,107	165,448,937	T_Width_Sum_SS	Whole plant leaf sheath
GRMZM2G339645	CSLF3—cellulose synthase-like family F	7	NC_050102.1	165,345,171	165,348,432	T_Width_Sum_SS	Whole plant leaf sheath
GRMZM2G066997	Remorin	5	NC_050100.1	193,077,446	193,080,950	T_Width_Sum_SS, T_Area_S0, T_LWRatio_S0	Whole plant and Sixth leaf sheath
GRMZM2G477314	CF9	1	NC_050096.1	288,099,406	288,101,023	Sixth_PC1	Sixth leaf sheath
GRMZM2G091303	Xyloglucan endotransglucosylase/hydrolase protein 24	10	NC_050105.1	143,472,231	143,474,095	Sixth_PC1	Sixth leaf sheath
CKX10	Cytokinin dehydrogenase 10	1	NC_050096.1	21,236,2957	212,366,306	Sixth_PC2	Sixth leaf sheath
GRMZM2G122126	6-Phosphogluconolactonase	1	NC_050096.1	212,272,609	212,274,483	Sixth_PC2	Sixth leaf sheath
GRMZM2G138355	Nudix hydrolase 13	10	NC_050105.1	114,632,712	114,636,142	Sixth_PC2	Sixth leaf sheath
Zm00001d027570	Putative protein phosphatase 2C 48	1	NC_050096.1	8,216,152	8,220,401	T_Area_S0	Sixth leaf sheath
GRMZM6G207008	Characterized LOC100272314	4	NC_050099.1	172,883,765	172,884,420	T_Compactness_ S0	Sixth leaf sheath
Zm00001d051817	DNA topoisomerase 2	4	NC_050099.1	172,603,028	172,604,370	T_Compactness_ S0	Sixth leaf sheath
GRMZM2G339907	NDR1/HIN1-like protein 26	7	NC_050102.1	163,429,440	163,430,393	T_LWRatio_S0	Sixth leaf sheath
GRMZM2G039811	Transmembrane 9 superfamily member 9	2	NC_050097.1	204,934,314	204,938,233	T_Perimeter_S0	Sixth leaf sheath
GRMZM2G153369	Hydrophobic protein RCI2B	2	NC_050097.1	205,006,561	205,007,668	T_Perimeter_S0	Sixth leaf sheath
GRMZM2G007122	Putative ubiquitin-conjugating enzyme family	6	NC_050101.1	175,737,238	175,740,387	T_Perimeter_S0	Sixth leaf sheath
GRMZM2G155686	Gibberellin 2-oxidase8	6	NC_050101.1	175,763,619	175,765,545	T_Perimeter_S0	Sixth leaf sheath

Hence, it was obvious that each leaf sheath-related trait had its own specific candidate gene, whether it was from the whole plant or the sixth leaf alone. In addition to the common traits that could be extracted in previous studies, the 2D leaf sheath-related traits proposed in this study also identified significant loci and candidate genes. Consequently, it is necessary to subdivide and refine the phenotype of plants at maize seedling stage (Table 1). In addition, some of these traits had overlapped genes in the whole plant and the sixth leaf sheath (Table 1), indicating that these traits were genetically related to a certain degree. If the study on the sixth leaf sheath can be used instead of the whole plant study at V6 stage, it will greatly save the cost of phenotype acquisition.

### Pathways Enriched by Functional Enrichment Analysis

**FIGURE 6:**
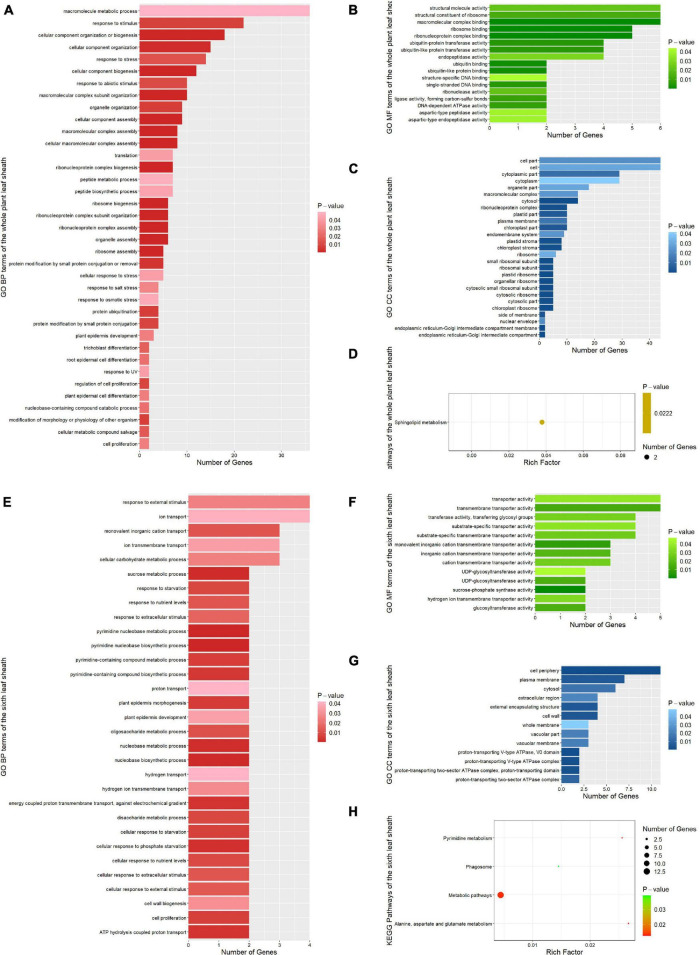
Functional enrichment results of all candidate genes associated with phenotypic traits. **(A)** GO BP (biological process) terms enriched by the whole plant leaf sheath candidate genes. **(B)** GO MF (molecular function) terms enriched by the whole plant leaf sheath candidate genes. **(C)** GO CC (cellular components) terms enriched by the whole plant leaf sheath candidate genes. **(D)** KEGG pathways enriched by the whole plant leaf sheath candidate genes. **(E)** GO BP terms enriched by the sixth leaf sheath candidate genes. **(F)** GO MF terms enriched by the sixth leaf sheath candidate genes. **(G)** GO CC terms enriched by the sixth leaf sheath candidate genes. **(H)** KEGG pathways enriched by the sixth leaf sheath candidate genes.

In order to further explore the function of candidate genes, we used functional enrichment analysis to enrich the candidate genes annotated by the most significant SNP (Top1) and verified by multiple methods in the whole plant and the sixth leaf sheath, respectively. For the whole plant leaf sheath traits, a total of 81 GO terms and 1 KEGG pathways (*P* < 0.05) were obtained by enrichment of candidate genes for leaf sheath phenotype, among which 37 GO terms belonged to GO BP (biological process) (Figures 6A–D). In GO BP terms, the pathways with the highest significance were related to cellular component assembly and organization. For instance, “ribosome assembly” (GO:0042255, *P*-value = 2.70E-09), “organelle assembly” (GO:0070925, *P*-value = 3.50E-09), “ribonucleoprotein complex assembly” (GO:0022618, *P*-value = 6.90E-08), “cellular macromolecular complex assembly” (GO:0034622, *P*-value = 1.30E-05), “cellular component assembly” (GO:0022607, *P*-value = 5.80E-05) and “cellular component organization or biogenesis” (GO:0071840, *P*-value = 0.00016). Notably, several pathways related to cell proliferation and epidermal cell differentiation were identified by GO analysis: “regulation of cell proliferation” (GO:0042127, *P*-value = 0.00834), “cell proliferation” (GO:0008283, *P*-value = 0.02853), “root epidermal cell differentiation” (GO:0010053, *P*-value = 0.02308), “plant epidermal cell differentiation” (GO:0090627, *P*-value = 0.02853) and “plant epidermis development” (GO:0090558, *P*-value = 0.02884). In addition, the one KEGG pathway was “Sphingolipid metabolism” (zma00600, *P*-value = 0.02218).

For the sixth leaf sheath traits, a total of 57 GO terms and 4 KEGG pathways (*P*-value < 0.05) were enriched in the sixth leaf sheath phenotype candidate genes, among which 31 GO terms belonged to GO BP (Figures 6E–H). In GO BP terms, several pathways related to response to hunger, nutrition and extracellular stimulation were enriched by genes *GRMZM2G147450* and *GRMZM2G059121*: “cellular response to phosphate starvation” (GO:0016036, *P*-value = 0.00245), “cellular response to starvation” (GO:0009267, *P*-value = 0.00643), “disaccharide metabolic process” (GO:0005984, *P*-value = 0.00779), “response to starvation” (GO:0042594, *P*-value = 0.00779), “cellular response to nutrient levels” (GO:0031669, *P*-value = 0.00842), “response to nutrient levels” (GO:0031667, *P*-value = 0.01184), “cellular response to extracellular stimulus” (GO:0031668, *P*-value = 0.01184) and “cellular response to external stimulus” (GO:0071496, *P*-value = 0.01284). In addition, candidate genes for the sixth leaf sheath traits were also enriched in multiple pathways related to cell proliferation and epidermis development. For example, “plant epidermis morphogenesis” (GO:0090626, *P*-value = 0.00519), “cell proliferation” (GO:0008283, *P*-value = 0.00606) and “plant epidermis development” (GO:0090558, *P*-value = 0.03493). The most striking result of KEGG is “Alanine, aspartate and glutamate metabolism” (zma00250, *P*-value = 0.01283). And the other three pathways are “Pyrimidine metabolism” (zma00240, *P*-value = 0.01379), “Metabolic pathways” (zma01100, *P*-value = 0.01382) and “Phagosome” (zma04145, *P*-value = 0.03899).

### Trait-Candidate Gene-Pathway Network Visualization

**FIGURE 7:**
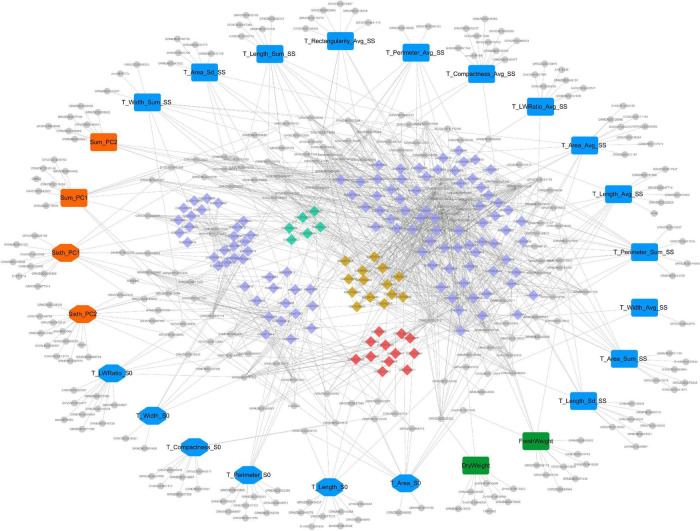
The “trait-gene-pathway” network constructed by 25 key traits and their candidate genes and pathways. Traits, genes and pathways (GO terms and KEGG pathways) are shown in different shapes and sizes. Of the 25 large nodes, 17 round rectangle nodes represent the whole plant leaf sheath traits, and 8 octagon nodes represent the sixth leaf sheath traits. And different color represents different type of traits (blue- morphology, orange- color and green- biomass). The colorful small diamonds represent GO terms and KEGG pathways enriched by candidate genes. Among them, pathways related to cellular component assembly and organization were marked in earthy yellow, pathways related to cell proliferation and epidermal cell differentiation were marked in grass green, and pathways related to response to hunger, nutrition and extracellular stimulation were marked in red. Candidate genes are represented by the small gray circular nodes.

Cytoscape V3.7.2 was used to draw the trait-candidate gene-pathway network of 2D maize leaf sheath traits at seedling stage, and to show the relationship between 270 candidate genes and 25 key traits, and between candidate genes and their enriched pathways. The whole network consisted of 444 nodes and 1,144 edges (Figure 7). In the network, there were 25 traits (the largest nodes), including 17 whole plant leaf sheath traits (round rectangle nodes) and 8 sixth leaf sheath traits (octagon nodes). And the types of traits—morphology, color and biomass—were also marked in blue, orange and green, respectively. In addition, the candidate genes were marked with small gray circular nodes, and the pathways were marked with small diamond. Among them, pathways related to cellular component assembly and organization were marked in earthy yellow, pathways related to cell proliferation and epidermal cell differentiation were marked in grass green, and pathways related to response to hunger, nutrition and extracellular stimulation were marked in red.

## Discussion

Maize leaf sheaths wrap stem to provide structural support and protect developing leaves, which is of great biological significance. This study broke the traditional method of phenotypic acquisition of maize leaf sheath, and proposed an image-based high-throughput acquisition and data analysis scheme for phenotypic traits of maize leaf sheath from image acquisition, image phenotypic analysis and leaf sheath phenotypic data analysis. Firstly, a simple and reliable environment for maize leaf sheath image acquisition was established, and the acquisition time of a single sample image was less than 10s. Then, a maize leaf sheath phenotypic image analysis software with friendly interactive interface was developed based on open-source software development tools. Based on the image analysis, 85 leaf sheath phenotypic traits including shape and color can be analyzed, and the calculation time for a single image was less than 60s. Finally, phenotypic traits were extracted and analyzed from leaf sheath images of 418 maize inbred lines, and the statistical description results of leaf sheath phenotypic traits of large maize populations were obtained. It is time-consuming and laborious to obtain the traditional traits such as length and width of leaf sheath manually, but the image-based phenotypic acquisition method can quickly obtain the length and width of leaf sheath in less than 1 min. Besides, more than 80 phenotypic traits can also be extracted. Thus, efficient and high-throughput acquisition of leaf sheath phenotypes was achieved. Moreover, this method is suitable for large populations and can help to obtain leaf sheath phenotype in maize association analysis population.

A large number of traits can be extracted from plant images, and a variety of new traits can be determined from different dimensions. However, the interpretability of the traits still needs further study. In this study, correlation analysis, cluster analysis and PCA were performed on 87 leaf sheath-related phenotypic traits of maize association analysis population. The results showed that there were differences in morphological characteristics and color traits of leaf sheath, with correlation coefficients less than 0.5. In the morphological characteristics of leaf sheath, it can be divided into three groups with definite significance due to the different features described. Color traits can be subdivided into three subsets with distinctive features. Therefore, although some traits cannot explain their biological significance by themselves, combined with trait grouping and its highly correlated traits, the phenotypic traits with less clear meanings can be characterized.

In order to verify the reliability of phenotypic acquisition from leaf sheath images, correlation analysis was conducted between dry and fresh weight of maize leaf sheath measured manually and leaf sheath morphological traits obtained from images. The results showed that 16 morphological characteristics of leaf sheath had a significant positive correlation with DryWeight and FreshWeight (*p*-value < 0.05), and clustered into the same group (Morphological Traits_Basic and Biomass Traits). This result was consistent with the prior knowledge, revealing the reliability of the data, and demonstrating that the various traits obtained from the image were meaningful. Moreover, among the color traits extracted from the image, 24 comprehensive color traits were divided into two groups, CIVE, CIVE_S, ExR and ExR_S mainly represent red, while the remaining comprehensive traits mainly represent blue and green. The clustering results based on the phenotypic data were consistent with the trait characteristics, which also showed the accuracy of the data extraction.

It can be seen from the results of this study that image-based high-throughput phenotypic acquisition techniques can obtain novel traits that breeders cannot evaluate through traditional methods, such as geometric and color traits described quantitatively. In this study, 88.51% (77/87) of leaf sheath-related phenotypic traits had heritability greater than 0.3, indicating that the formation of these phenotypes was influenced by genetic factors. To further dissect the genetic mechanisms underlying these phenotypes with heritability greater than 0.3, GWAS was used to analyze the 25 key leaf sheath-related traits, and totally 3,113 candidate genes for leaf sheath-related traits were obtained. The candidate genes with high significance or verified by multiple methods were considered as high reliability results, which would provide reference for subsequent functional verification of maize leaf sheath candidate genes. For example, cytokinin dehydrogenase 10 (*CKX10*) is a candidate gene for major component traits of the color of the sixth leaf sheath (Sixth_PC2). Meanwhile, it has been reported that *CKX10* plays an important role in dry matter accumulation in V6 stage leaves (Lu et al., 2020). *CKX10* is a member of the CKX family, and a great deal of work has been done on this gene family in gramineae (Mameaux et al., 2012), including some studies on maize. In transcriptome analysis of maize, *CKX10* has also been reported as one of the DEGs of KEGG pathways associated with hormone metabolism (Zheng et al., 2020). Therefore, we speculate that *CKX10* plays an important role in the formation of leaf sheath color in maize V6 stage. It is worth noting that some loci of these high confidence results had a high explanatory power (PVE > 5%) for phenotypic variation. For example, *GRMZM2G135770*, putative regulator of chromosome condensation (RCC1) family protein, was annotated by chr4.S_84970911 on chromosome 4, which was significantly associated with the trait T_Length_Sum_SS, and explained 6.54% of the phenotypic variance. *GRMZM2G156238*, C2 domain-containing protein At1g53590, which has been proved to be tissue-specific (Stelpflug et al., 2016). It was reported in the study of organ-specific and stress-induced gene expression mapping of maize (Hoopes et al., 2019). In this study, it was annotated by chr4.S_224037650, also located on chromosome 4, which was significantly associated with the trait T_Area_Avg_SS, explaining 5.17% of the phenotypic variance. And *GRMZM2G156238* was also associated with the other two leaf sheath morphological traits (T_Area_S0 and T_Perimeter_Avg_SS). The above results proved the reliability of the phenotype-genotype association analysis process and results of this study. At the same time, it also reflects the significance of trait refinement for the research of crop phenotypic genetics, that is, the more refined the trait, the stronger the phenotype interpretability of the obtained locus.

Pigment plays an important role in plant reproduction and adaptability, and the research on plant pigment has always been a hot topic. In this study, phenotypic traits of leaf sheath color of maize inbred lines from four subpopulations with different environmental adaptability were analyzed. The results showed that there were significant differences in 48 leaf sheath color traits between tropical and subtropical maize inbred lines (TST) and maize inbred lines from other climatic zones (*P*-value < 0.05), which showed that the color of maize leaf sheath was closely related to the ecological adaptability and evolution of maize. In addition, the changes of pigment deposition, distribution and shade among different kinds of maize are of great value to the study of maize functional genome and the application of maize genetics and breeding. Leaf sheath color is also an important morphological marker to guide maize breeding. It can be used for more intuitive selection and more directly genetic research of related special traits. In this study, a total of 60 leaf sheath color traits were extracted based on images, including 30 for the whole plant leaf sheath and 30 for the sixth leaf sheath. In addition to simple single-channel color traits, a number of novel comprehensive color traits were also extracted. The results of heritability analysis showed that the heritability of color trait was generally high, so it was necessary to conduct GWAS analysis to explore the genetic factors behind these traits. In our study, PCA was used to reduce the dimensionality of the color traits with heritability greater than 0.3, and then the first two principal components were selected for GWAS. As a consequence, more than 800 candidate genes related to color traits were identified (Table 1). These results greatly enrich the existing research results on maize leaf sheath genetics and provide a theoretical basis for better explaining the mechanism of maize leaf sheath phenotype formation.

In recent years, phenomics has emerged as a rapidly growing data-intensive discipline. The rapid development of phenomics-related technologies and research tools has brought about a huge amount of phenotypic information at multiple scales and data diversity, such as RGB, hyperspectral, near-infrared, thermal and fluorescence imaging and other image data, as well as data on various physiological traits during plant growth (Kim et al., 2017). Crop life activity is a dynamic process under the combined action of genes and environment. As high-throughput sequencing technologies continue to develop and improve, single-omics studies are becoming increasingly sophisticated. And the integration of multi-omics data to study crops is on the rise. Genomic studies combining genomic and phenotypic data have been conducted in many crops and have rapidly decoded the functions of a large number of unknown genes. In 2014, 13 traditional agronomic traits of rice were combined with two newly defined traits and 141 related loci were identified using GWAS (Yang W. et al., 2014). In 2015, 29 leaf phenotypic traits at three key fertility stages were resolved using high-throughput leaf phenotype acquisition (HLS) and subjected to GWAS analysis, and 73 loci regulating leaf size, 123 loci regulating leaf color and 177 new loci regulating leaf shape (Yang et al., 2015). In 2021, 48 maize stem micro-phenotypic traits were automatically extracted by micro-CT image processing pipeline and 1,562 significant SNPs were identified for 30 key traits by GWAS (Zhang et al., 2021). It is clear that combining high-throughput phenotyping techniques with large-scale QTL or GWAS analysis not only greatly expands our understanding of the dynamic developmental processes in crops, but also provides a new tool for plant genomics, gene characterization and breeding research.

## Data Availability Statement

The datasets presented in this study can be found in online repositories. The names of the repository/repositories and accession number(s) can be found in the article/Supplementary Material.

## Author Contributions

XG and WS conceived and supervised the project and agreed to serve as the author responsible for contact and ensure communication. XL, YZ, YaZ, and WW conducted the experiment and collected the data. JW and CW analyzed data, prepared figures, wrote the manuscript, and drafted and revised the manuscript. All authors contributed to the article and approved the submitted version.

## Conflict of Interest

The authors declare that the research was conducted in the absence of any commercial or financial relationships that could be construed as a potential conflict of interest.

## Publisher’s Note

All claims expressed in this article are solely those of the authors and do not necessarily represent those of their affiliated organizations, or those of the publisher, the editors and the reviewers. Any product that may be evaluated in this article, or claim that may be made by its manufacturer, is not guaranteed or endorsed by the publisher.

## Footnotes

1
https://cran.r-project.org/

2
www.maizego.org/Resources.html

3
http://plants.ensembl.org/Zea_mays/Info/Traits

4
https://www.ncbi.nlm.nih.gov/gene

## Supplementary Material

Supplementary Figure 1 The trait variation between subpopulations (TST, NSS, SS, and mixed) for the 87 phenotypic traits (divided into three types).

Supplementary Figure 2 Sample grouping results based on **(A)** 21 leaf sheath color traits of whole plant, **(B)** 25 leaf sheath color traits of the sixth leaf and **(C)** 4 leaf sheath morphological traits, respectively.

Supplementary Table 3 Descriptive statistical analysis results of 87 maize leaf sheath phenotypic traits.

Supplementary Table 4 270 candidate genes of 25 key traits for leaf sheath phenotype with detailed functional descriptions from NCBI Gene database.
