# Supplementary figures and images for: Dissecting the Genetic Structure of Maize Leaf Sheaths at Seedling Stage by Image-Based High-Throughput Phenotypic Acquisition and Characterization
Source: Front Plant Sci. 2022 Jun 28;13:826875. doi: 10.3389/fpls.2022.826875 (PMC9274118; doi:10.3389/fpls.2022.826875)

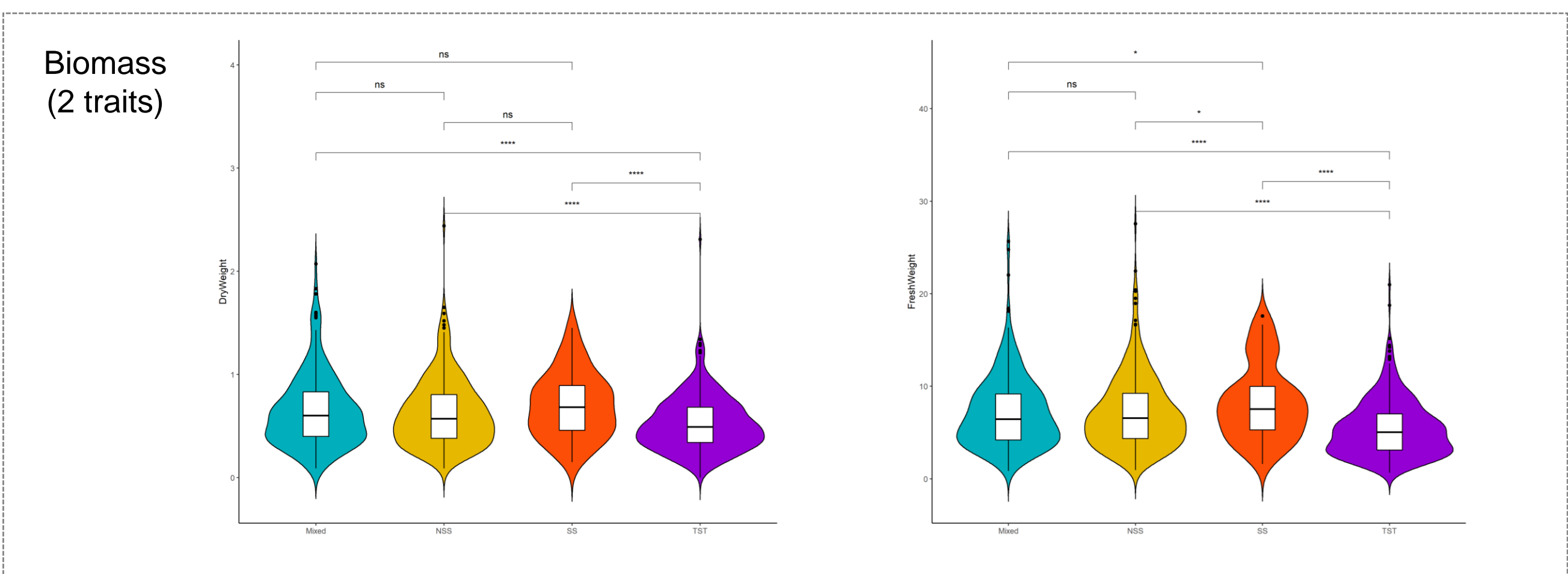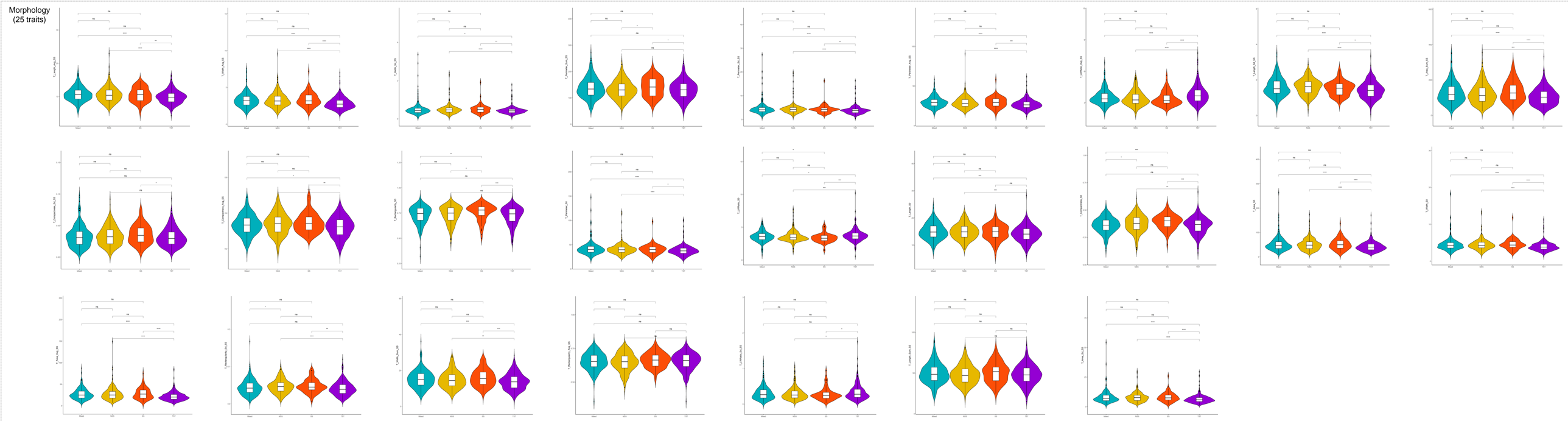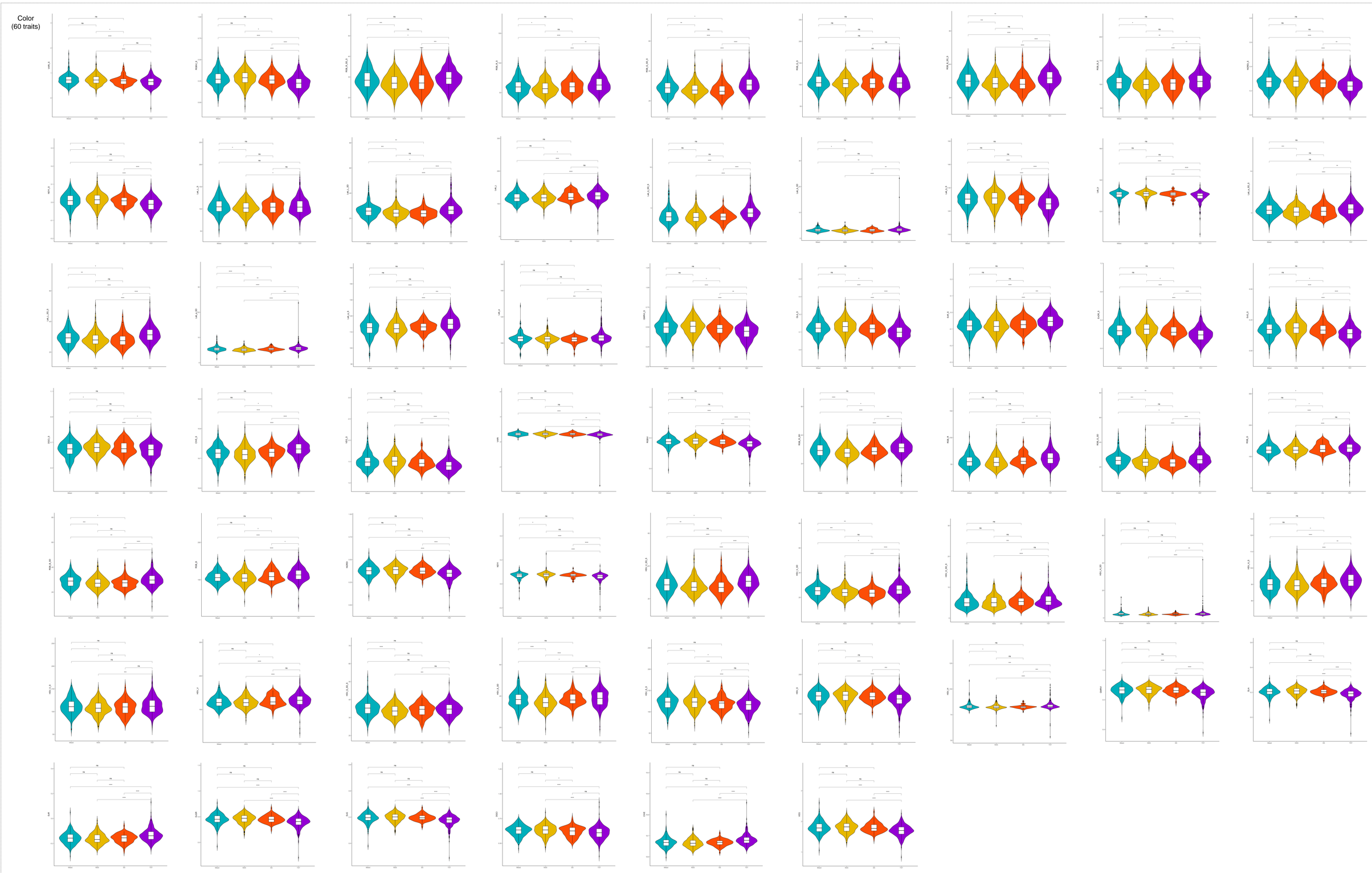

Supplement: Supplementary Figure 1 — The trait variation between subpopulations (TST, NSS, SS, and mixed) for the 87 phenotypic traits (divided into three types). [file Image_1.PDF]

A

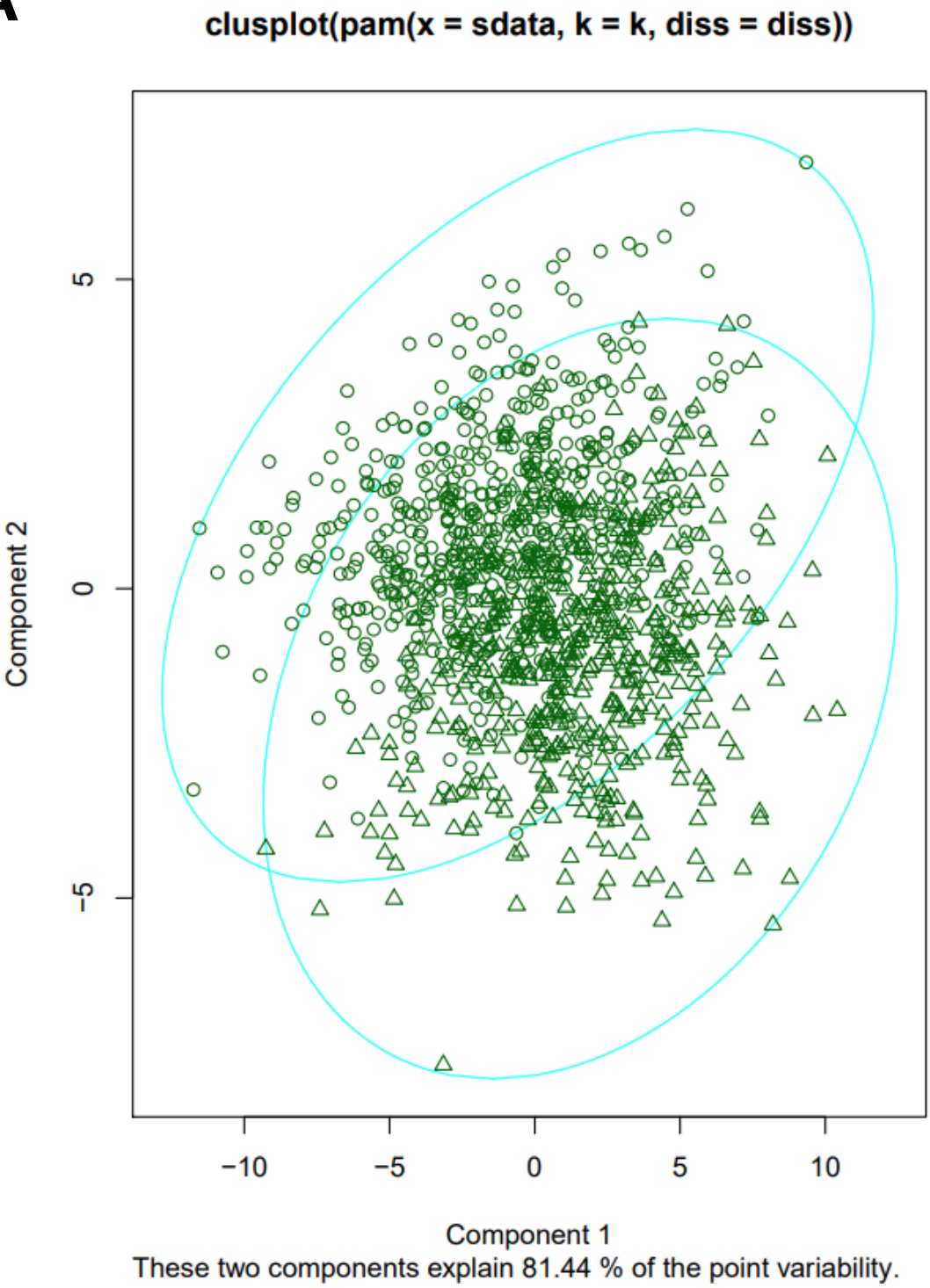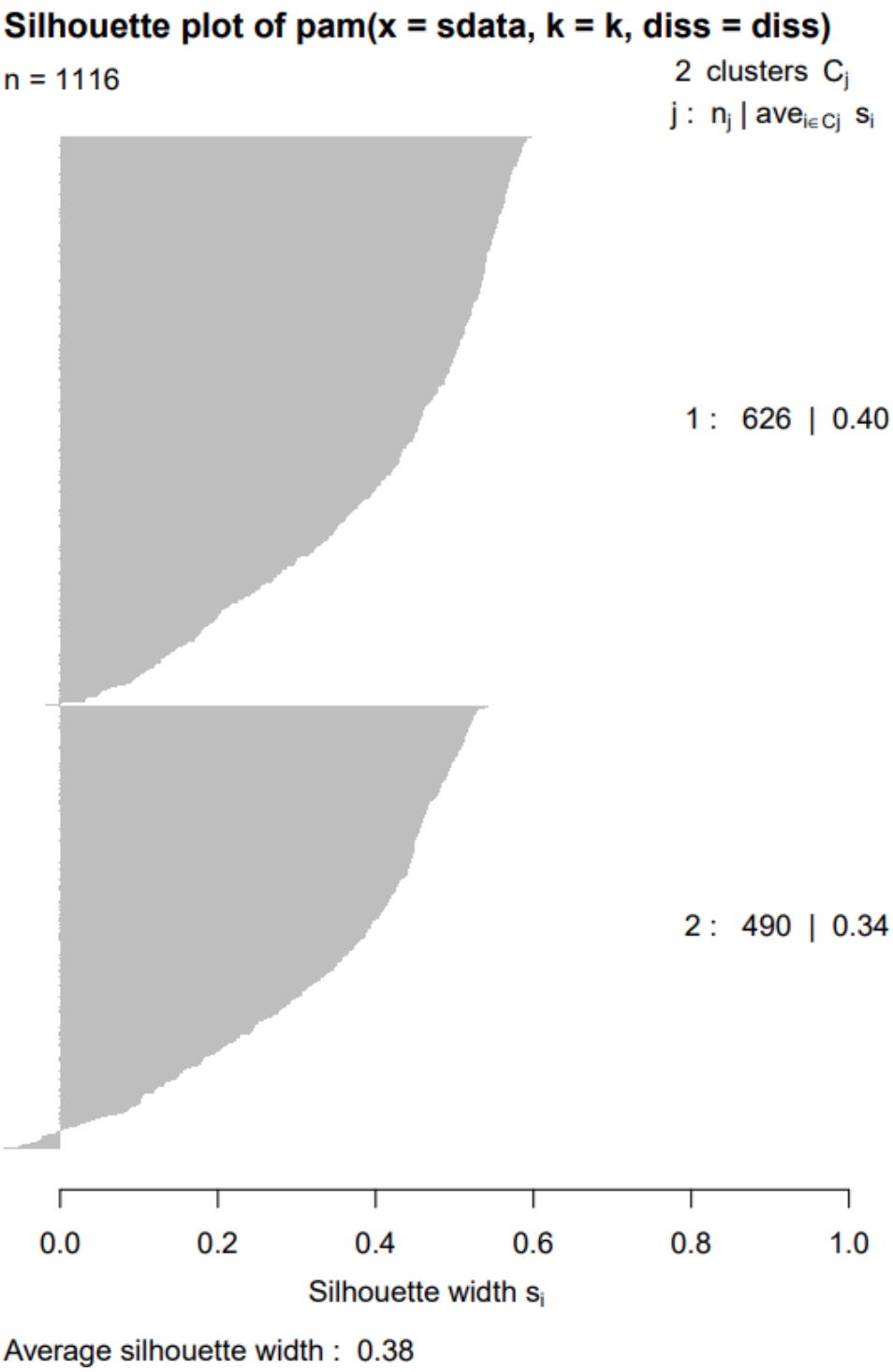

B

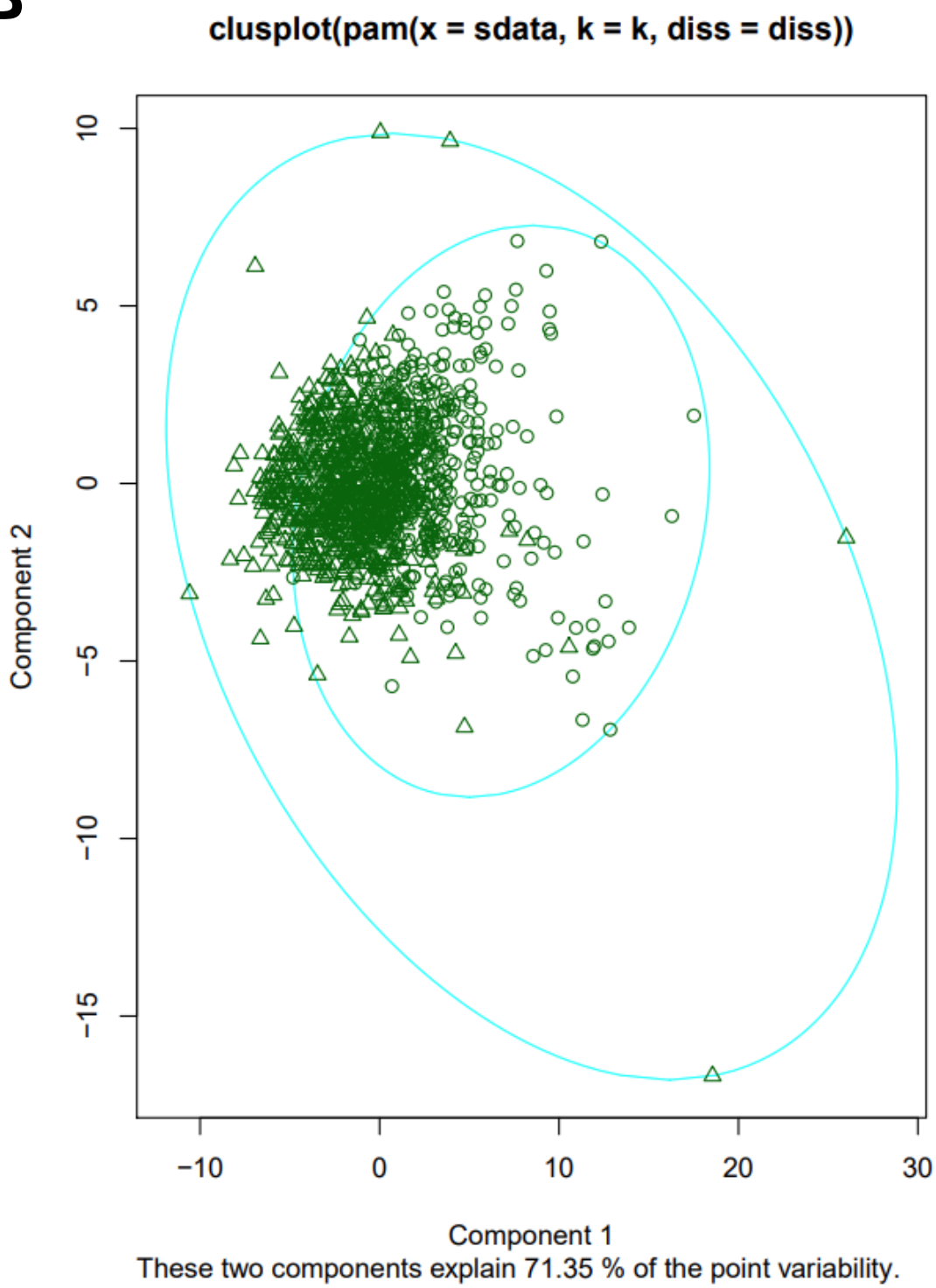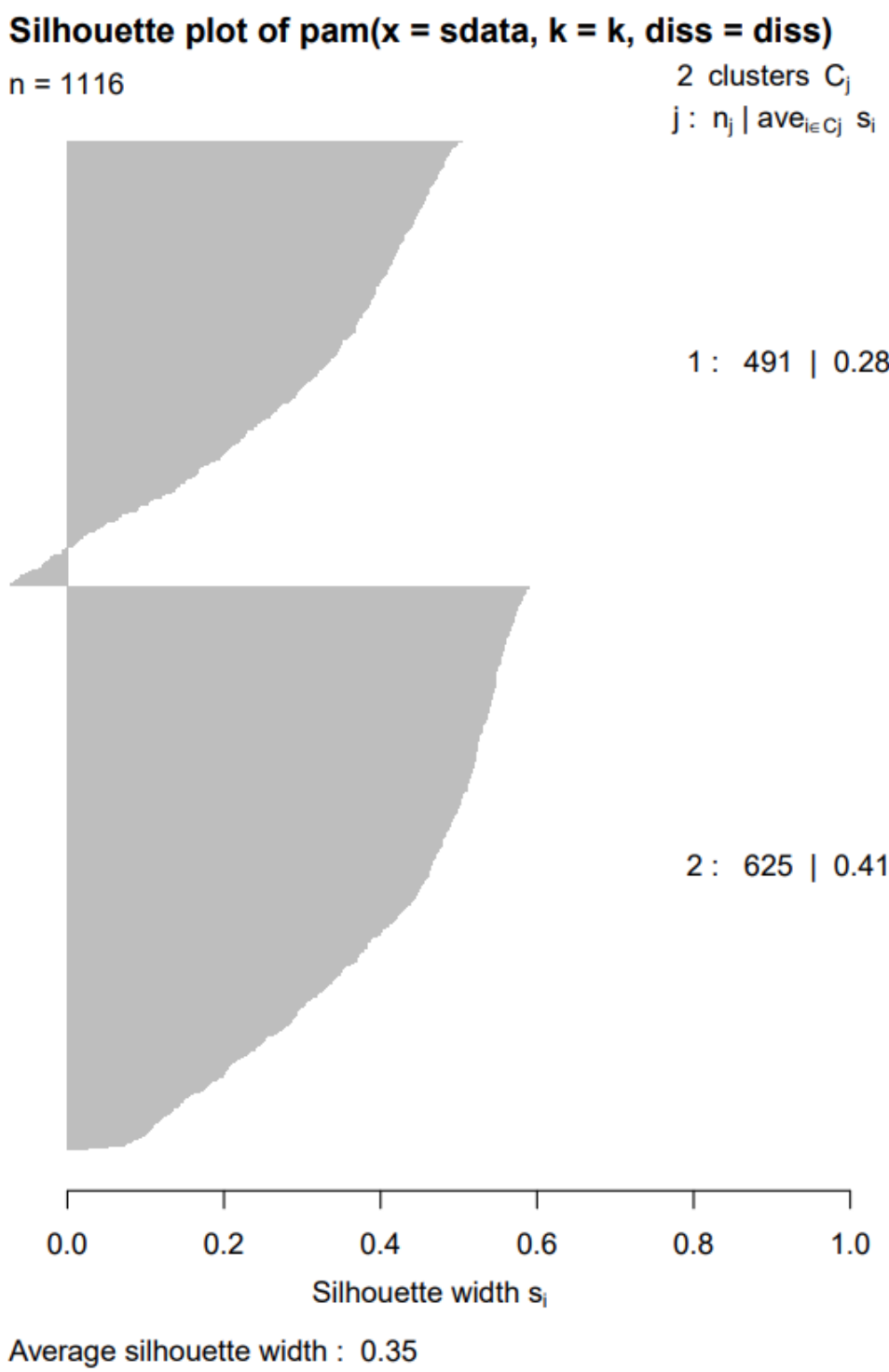

C

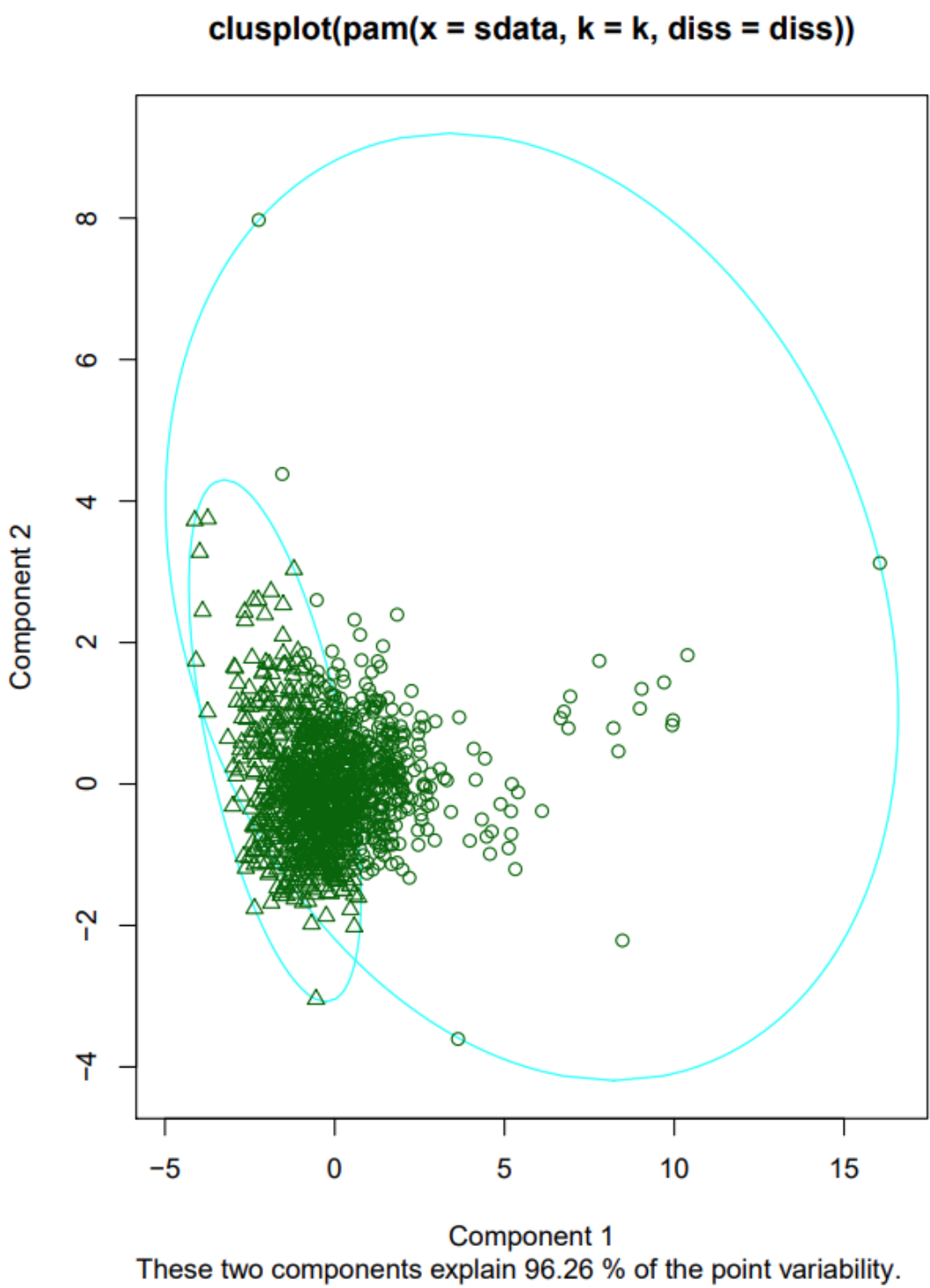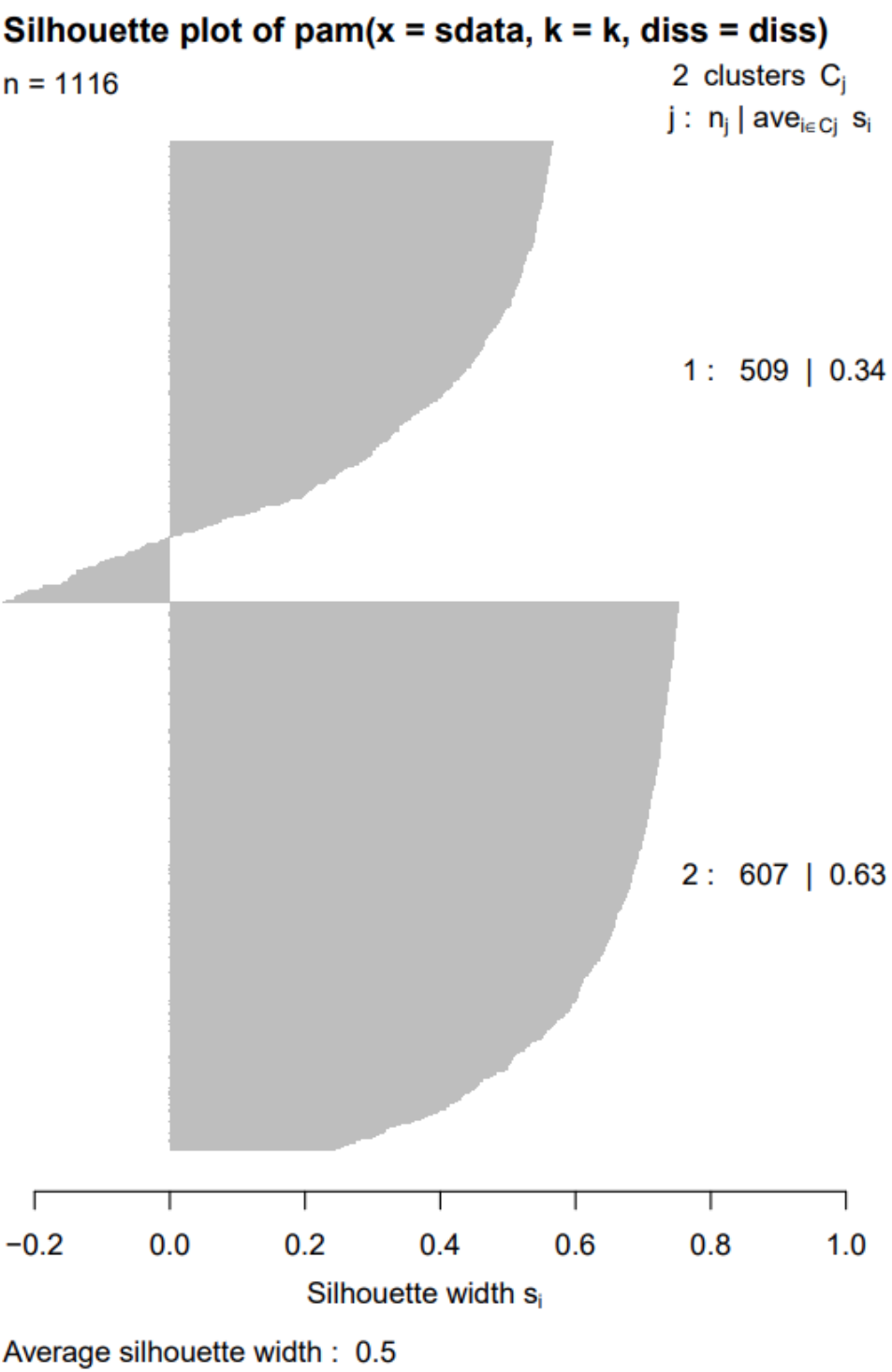

Supplement: Supplementary Figure 2 — Sample grouping results based on (A) 21 leaf sheath color traits of whole plant, (B) 25 leaf sheath color traits of the sixth leaf and (C) 4 leaf sheath morphological traits, respectively. [file Image_2.PDF]
