# Supplementary material for: Dissecting the Genetic Structure of Maize Leaf Sheaths at Seedling Stage by Image-Based High-Throughput Phenotypic Acquisition and Characterization
Source: Front Plant Sci. 2022 Jun 28;13:826875. doi: 10.3389/fpls.2022.826875 (PMC9274118; doi:10.3389/fpls.2022.826875)
Supplement: Supplementary file 4 [file Table_2.PDF]

**Supplementary Table 2** Definition and description of 87 phenotypic traits based on images and manual measurements in this study. More details about the traits' extraction can be found in the Supplementary Note 1.

| Category | Classification | Description | Object | Trait | Unit |
|---|---|---|---|---|---|
| Biomass | Dry weight | Dry weight of the whole plant at V6 stage | Whole Plant | DryWeight | g |
| | Fresh weight | Fresh weight of the whole plant at V6 stage | | FreshWeight | g |
| Morphology | Length | Average leaf sheath length of all expanded leaves | Whole Plant | T_Length_Avg_SS | mm |
| | | Standard deviation of leaf sheath length of all expanded leaves | | T_Length_Sd_SS | mm |
| | | Summation leaf sheath length of all expanded leaves | | T_Length_Sum_SS | mm |
| | | Length of the sixth expanded leaf sheath | Sixth Leaf | T_Length_S0 | mm |
| | Width | Average leaf sheath width of all expanded leaves | Whole Plant | T_Width_Avg_SS | mm |
| | | Standard deviation of leaf sheath width of all expanded leaves | | T_Width_Sd_SS | mm |
| | | Summation leaf sheath width of all expanded leaves | | T_Width_Sum_SS | mm |
| | | Width of the sixth expanded leaf sheath | Sixth Leaf | T_Width_S0 | mm |
| | Perimeter | Average leaf sheath perimeter of all expanded leaves | Whole Plant | T_Perimeter_Avg_SS | mm |

| | | | | |
|---|---|---|---|---|
| | Standard deviation of leaf sheath perimeter of all expanded leaves | | T_Perimeter_Sd_SS | mm |
| | Summation leaf sheath perimeter of all expanded leaves | | T_Perimeter_Sum_SS | mm |
| | Perimeter of the sixth expanded leaf sheath | Sixth Leaf | T_Perimeter_S0 | mm |
| Area | Average leaf sheath area of all expanded leaves | Whole Plant | T_Area_Avg_SS | $mm^2$ |
| | Standard deviation of leaf sheath area of all expanded leaves | | T_Area_Sd_SS | $mm^2$ |
| | Summation leaf sheath area of all expanded leaves | | T_Area_Sum_SS | $mm^2$ |
| | Area of the sixth expanded leaf sheath | Sixth Leaf | T_Area_S0 | $mm^2$ |
| LWRatio | Average leaf sheath length-width ratio of all expanded leaves | Whole Plant | T_LWRatio_Avg_SS | - |
| | Standard deviation of leaf sheath area of all expanded leaves | | T_LWRatio_Sd_SS | - |
| | Standard deviation of the sixth expanded leaf sheath | Sixth Leaf | T_LWRatio_S0 | - |
| Compactness | Average leaf sheath compactness of all expanded leaves | Whole Plant | T_Compactness_Avg_SS | - |
| | Standard deviation of leaf sheath compactness of all expanded leaves | | T_Compactness_Sd_SS | - |
| | Compactness of the sixth expanded leaf sheath | Sixth Leaf | T_Compactness_S0 | - |
| Rectangularity | Average rectangularity of all expanded leave sheaths | Whole Plant | T_Rectangularity_Avg_SS | - |

| | | | | | |
|---|---|---|---|---|---|
| | | Standard deviation of rectangularity of all expanded leave sheaths | | T_Rectangularity_Sd_SS | - |
| | | Rectangularity of the sixth expanded leaf sheath | Sixth Leaf | T_Rectangularity_S0 | - |
| Colour | Integrated traits | Color channel combination value | Whole Plant | CIVE_S | - |
| | | | Sixth Leaf | CIVE | - |
| | | | Whole Plant | DGCI_S | - |
| | | | Sixth Leaf | DGCI | - |
| | | | Whole Plant | ExGR_S | - |
| | | | Sixth Leaf | ExGR | - |
| | | | Whole Plant | ExG_S | - |
| | | | Sixth Leaf | ExG | - |
| | | | Whole Plant | ExR_S | - |
| | | | Sixth Leaf | ExR | - |
| | | | Whole Plant | GLA_S | - |
| | | | Sixth Leaf | GLA | - |

| | | | | |
|---|---|---|---|---|
| | | Whole Plant | GMRVI_S | - |
| | | Sixth Leaf | GMRVI | - |
| | | Whole Plant | NDYI_S | - |
| | | Sixth Leaf | NDYI | - |
| | | Whole Plant | NGRDI_S | - |
| | | Sixth Leaf | NGRDI | - |
| | | Whole Plant | RGBVI_S | - |
| | | Sixth Leaf | RGBVI | - |
| | | Whole Plant | VARI_S | - |
| | | Sixth Leaf | VARI | - |
| | | Whole Plant | VEG_S | - |
| | | Sixth Leaf | VEG | - |
| Single channel value of color space | Standard deviation of R channel value of RGB color space | Whole Plant | RGB_R_SD_S | - |
| | | Sixth Leaf | RGB_R_SD | - |

| | | | |
|---|---|---|---|
| Average R channel value of RGB color space | Whole Plant | RGB_R_S | - |
| | Sixth Leaf | RGB_R | - |
| Standard deviation of G channel value of RGB color space | Whole Plant | RGB_G_SD_S | - |
| | Sixth Leaf | RGB_G_SD | - |
| Average G channel value of RGB color space of the sixth expanded leaf image pixels | Whole Plant | RGB_G_S | - |
| | Sixth Leaf | RGB_G | - |
| Standard deviation of B channel value of RGB color space | Whole Plant | RGB_B_SD_S | - |
| | Sixth Leaf | RGB_B_SD | - |
| Average B channel value of RGB color space | Whole Plant | RGB_B_S | - |
| | Sixth Leaf | RGB_B | - |
| Standard deviation of L channel value of Lab color space | Whole Plant | Lab_L_SD_S | - |
| | Sixth Leaf | Lab_L_SD | - |
| Average L channel value of Lab color space | Whole Plant | Lab_L_S | - |
| | Sixth Leaf | Lab_L | - |

| | | | |
|---|---|---|---|
| Standard deviation of b channel value of Lab color space | Whole Plant | Lab_b_SD_S | - |
| | Sixth Leaf | Lab_b_SD | - |
| Average b channel value of Lab color space | Whole Plant | Lab_b_S | - |
| | Sixth Leaf | Lab_b | - |
| Standard deviation of a channel value of Lab color space | Whole Plant | Lab_a_SD_S | - |
| | Sixth Leaf | Lab_a_SD | - |
| Average a channel value of Lab color space | Whole Plant | Lab_a_S | - |
| | Sixth Leaf | Lab_a | - |
| Standard deviation of V channel value of HSV color space | Whole Plant | HSV_V_SD_S | - |
| | Sixth Leaf | HSV_V_SD | - |
| Average V channel value of HSV color space | Whole Plant | HSV_V_S | - |
| | Sixth Leaf | HSV_V | - |
| Standard deviation of S channel value of HSV color space | Whole Plant | HSV_S_SD_S | - |
| | Sixth Leaf | HSV_S_SD | - |

| | | | | |
|---|---|---|---|---|
| | Average S channel value of HSV color space | Whole Plant | HSV_S_S | - |
| | | Sixth Leaf | HSV_S | - |
| | Standard deviation of H channel value of HSV color space | Whole Plant | HSV_H_SD_S | - |
| | | Sixth Leaf | HSV_H_SD | - |
| | Average H channel value of HSV color space | Whole Plant | HSV_H_S | - |
| | | Sixth Leaf | HSV_H | - |
| Principal component | The first principal component obtained by principal component analysis (PCA) of 30 color traits. | Whole Plant | Sum_PC1 | - |
| | | Sixth Leaf | Sixth_PC1 | - |
| | The second principal component obtained by principal component analysis (PCA) of 30 color traits. | Whole Plant | Sum_PC2 | - |
| | | Sixth Leaf | Sixth_PC2 | - |
| | The third principal component obtained by principal component analysis (PCA) of 30 color traits. | Whole Plant | Sum_PC3 | - |
| | | Sixth Leaf | Sixth_PC3 | - |

**Supplementary Note 1.** Definition of the traits

①The 3 color space RGB, HSV and Lab, each color space contains 3 channels, calculate the average and standard deviation value of color channel of leaf sheath image pixels, resulted 18 traits from all leaf sheaths image, resulted 18 traits from the sixth leaf sheath.

②Separation of the leaf sheath image into red (R), green (G), and blue (B) color channels, the normalization color channels $r$, $g$ and $b$ were calculated as follows:

$$r = \frac{R}{R+G+B} \qquad (1)$$

$$g = \frac{G}{R+G+B} \qquad (2)$$

$$b = \frac{B}{R+G+B} \qquad (3)$$

Then the CIVE was calculated using the following equation:

$$CIVE = 0.441r - 0.811g + 0.385b + 18.78745 \qquad (4)$$

The DGCI was calculated using the following equation:

$$DGCI = \frac{(H-60)/60+(1-S)+(1-I)}{3} \qquad (5)$$

Where H,S and I were the channels of HSI color space.

The ExGR was calculated using the following equation:

$$ExGR = 3g - 2.4r - b \qquad (6)$$

The ExG was calculated using the following equation:

$$ExG = 2g - r - b \qquad (7)$$

The ExR was calculated using the following equation:

$$ExR = 1.4r - g \qquad (8)$$

The GLA was calculated using the following equation:

$$GLA = (2G - R - B)/(2G + R + B) \quad (9)$$

The NDYI was calculated using the following equation:

$$NDYI = (G - B)/(G + B) \quad (10)$$

The NGRDI was calculated using the following equation:

$$NGRDI = (G - R)/(G + R) \quad (11)$$

The RGBVI was calculated using the following equation:

$$RGBVI = \frac{(g^2 - br)}{(g^2 + br)} \quad (12)$$

The GMRVI was calculated using the following equation:

$$GMRVI = \frac{(g^2 - r^2)}{(g^2 + r^2)} \quad (13)$$

The VARI was calculated using the following equation:

$$VARI = \frac{G - R}{G + R - B} \quad (14)$$

The VEG was calculated using the following equation:

$$VEG = \frac{g}{r^{0.667} b^{0.333}} \quad (15)$$

From equation (4) to (15) 12 traits were calculated from all leaf sheath image, 12 traits were calculated from the sixth leaf sheath image.

③T_Area_S0: With the whole binary image, the sixth leaf sheath area was extracted and the T_Area_S0 was calculated as the summation of the foreground pixels. The T_Area_Avg_SS was calculated as mean of all leaf sheath areas, The T_Area_Sd_SS was calculated as standard deviation of all leaf sheath areas,

The T_Area_Sum_SS was calculated as summation of all leaf sheath areas.

④T_Perimeter_S0: With the whole binary image, the contour of the sixth leaf sheath image was extracted and the perimeter was calculated as the summation of the contour pixels. The T_Perimeter_Avg_SS was calculated as mean of all leaf sheath perimeters, The T_Perimeter_Sd_SS was calculated as standard deviation of all leaf sheath perimeters, The T_Perimeter_Sum_SS was calculated as summation of all leaf sheath perimeters.

⑤T_Length_S0: referred to length of sixth leaf sheath bounding box. The T_Length_Avg_SS was calculated as mean of all leaf sheath lengths, T_Length_Sd_SS was calculated as standard deviation of all leaf sheath lengths, T_Length_Sum_SS was calculated as summation of all leaf sheath lengths.

⑥T_Width_S0: referred to width of sixth leaf sheath bounding box. The T_Width_Avg_SS was calculated as mean of all leaf sheath widths, T_Width_Sd_SS was calculated as standard deviation of all leaf sheath widths, T_Width_Sum_SS was calculated as summation of all leaf sheath widths.

⑦T_LWRatio_S0: With the whole binary image T_LWRatio_S0 was defined as length/width of the sixth leaf sheath image. T_LWRatio_Avg_SS was calculated as mean of all leaf sheaths length/width, T_LWRatio_Sd_SS was calculated as standard deviation of all leaf sheaths length/width.

⑧T_Compactness_S0: With the whole binary image, T_Compactness_S0 was defined as compactness of the sixth leaf sheaths image.

$$compactness = \frac{A_l}{A_c} \qquad (16)$$

Where $A_l$ is the area of leaf sheaths binary image, $A_c$ is the area of convex hull of leaf sheaths binary image.

⑨T_Rectangularity_S0: With the whole binary image, T_Rectangularity_S0 was defined as rectangularity of the sixth leaf sheaths image.

$$rectangularity = \frac{A_l}{A_t} \qquad (17)$$

Where $A_l$ is the area of leaf sheaths binary image, $A_t$ is the area of bounding box of leaf lamina binary image.
